# Oil and Gas Wells and Pipelines on U.S. Wildlife Refuges: Challenges for Managers

**DOI:** 10.1371/journal.pone.0124085

**Published:** 2015-04-27

**Authors:** Pedro Ramirez, Sherri Baker Mosley

**Affiliations:** 1 NWRS, Division of Natural Resources and Conservation, U.S. Fish and Wildlife Service, Fort Collins, Colorado, United States of America; 2 NWRS, Natural Resource Program Center, U.S. Fish and Wildlife Service, Fort Collins, Colorado, United States of America; University of Kansas, UNITED STATES

## Abstract

The increased demand for oil and gas places a burden on lands set aside for natural resource conservation. Oil and gas development alters the environment locally and on a much broader spatial scale depending on the intensity and extent of mineral resource extraction. The current increase in oil and gas exploration and production in the United States prompted an update of the number of pipelines and wells associated with oil and gas production on National Wildlife Refuge System (NWRS) lands. We obtained geospatial data on the location of oil and gas wells and pipelines within and close to the boundaries of NWRS lands (units) acquired as fee simple (*i*.*e*. absolute title to the surface land) by the U.S. Fish and Wildlife Service. We found that 5,002 wells are located in 107 NWRS units and 595 pipelines transect 149 of the 599 NWRS units. Almost half of the wells (2,196) were inactive, one-third (1,665) were active, and the remainder of the wells were either plugged and abandoned or the status was unknown. Pipelines crossed a total of 2,155 kilometers (1,339 miles) of NWRS fee simple lands. The high level of oil and gas activity warrants follow up assessments for wells lacking information on production type or well status with emphasis on verifying the well status and identifying abandoned and unplugged wells. NWRS fee simple lands should also be assessed for impacts from brine, oil and other hydrocarbon spills, as well as habitat alteration associated with oil and gas, including the identification of abandoned oil and gas facilities requiring equipment removal and site restoration.

## Introduction

Although the mission of the U.S. Fish and Wildlife Service’s (USFWS) National Wildlife Refuge System (NWRS) is to manage a national network of lands and waters for the primary purpose of wildlife conservation, oil and gas wells and pipelines exist on many NWRS lands with privately owned subsurface minerals. These non-federal mineral rights remained in private ownership when the USFWS acquired the lands for inclusion into the NWRS. The owners of subsurface minerals or “mineral rights” have the legal right to explore for and extract oil and gas from their mineral estate. The increased demand for oil and gas and the extraction of these resources both globally and nationally places a burden on lands set aside for natural resource conservation. The United States Energy Information Administration (EIA) projects global energy demands to increase by 56 percent between 2010 and 2040 with natural gas consumption increasing by almost 2 percent each year [1]. In the U.S, the EIA [2] projects natural gas consumption to increase by 15 percent in the next 30 years with increasing use of this fuel in the generation of electricity. Bentley [3] forecasts a worldwide decline in the conventional production of oil and natural gas and an increase in the development of unconventional sources of oil and gas such as tar sands, shale gas, and shale oil. Exploration and production of oil and natural gas in the United States have increased significantly in the last decade, primarily due to technological advancements in horizontal drilling techniques and hydraulic fracturing coupled with the goal of reducing dependence on imported oil by one-third by 2025 [2, 4, 5, 6]. Annual petroleum production in the United States increased by 55 percent for oil and 74 percent for natural gas from 2003 to 2010 [7].

Oil and gas impacts on wildlife and the environment include habitat loss, wildlife mortality and displacement, and introduction of invasive species [8, 9, 10]. McDonald et al. [11] estimated the areal extent of impacts from oil and gas development between 5.7 to 32.4 hectares (ha) per well. Improper operation and maintenance of oil and gas activities can result in significant environmental impacts and injury to wildlife through spills, chronic leaks, and releases of crude oil, other hydrocarbons, and brine. Small releases of oil as well as exposed oil in open-topped tanks or pits can cause injury to wildlife and result in mortality [12, 13]. Fisher and Sublette [14] reported 12,863 spills and releases of liquids from oil and gas sites in Oklahoma during a 10-year period. Despite the fact that most spills were small in the amount of fluid released (median = 10 barrels (bbls)), environmental injury was still detected [14]. Small and repeated releases of oilfield brine kill vegetation and causes long-term damage to soils and wildlife habitat [15, 16]. The construction of roads and well pads to access and extract oil and gas results in the fragmentation and loss of wildlife habitat [17, 18, 19]. Wildlife will avoid areas of oil and gas development due to human activity and noise from equipment and vehicles [18, 20, 21, 22]. Oil and gas development also provides a mechanism for the spread of non-native vegetation, compromising ecological integrity [23].

In the United States, oil and gas development occurs on over 4.8 million ha of federally owned public land [24]. These federally owned lands provide a multitude of ecosystem services including recreation, wildlife protection, water supply, timber production, grazing, and mineral resources which contributed over $370 billion to the United States economy in 2012 [25]. Over 46 million people visited national wildlife refuges (NWRs) in 2011, contributing $2.4 billion to the economy and resulting in over $792 million in incomes for local communities [26]. Fee simple lands (*i*.*e*. absolute title to the surface land) in the NWRS include wildlife refuges, wildlife ranges, wildlife management areas, game preserves and conservation areas and waterfowl production areas. Waterfowl production areas are grouped by county and administered by USFWS wetland management districts. We use the term “NWRS unit” to refer to fee simple lands (*i*.*e*. absolute title to the surface land) within the NWRS, including lands withdrawn from public domain and included in the NWRS, as well as properties where the USFWS holds a secondary interest to another federal agency (overlay refuge). For example, the federal government established Hagerman NWR in Texas in 1946 on lands originally purchased by the U.S. Department of the Army Corps of Engineers for the Denison Dam Project (Lake Texoma).

The U.S. General Accounting Office (GAO) found that “*Federal management and oversight of oil and gas activities varies widely among refuges-some refuges take extensive measures*, *while others exercise little control or enforcement*” [27]. The GAO [27] attributed this variation in oversight and management of oil and gas activities to the lack of guidance, insufficient resources, and little or no training for refuge managers. The GAO [27] found that refuges also varied in how extensively they identified risks from oil and gas activities and how they managed those risks to minimize impacts. The GAO defined “refuge” as any unit of the NWRS, including national wildlife refuges, wildlife ranges, wildlife management areas, and waterfowl production areas (grouped by wetland management district). The GAO [27] reported 4,406 oil and gas wells (wells) in 105 refuges with the most wells located in refuges in Louisiana and Texas and only 3 refuges with NWRS staff dedicated to the oversight of oil and gas activities. Over half of the wells were inactive (plugged and abandoned, shut-in or temporarily idle, or dry holes). Forty-one percent of the wells were active and located in 36 refuges. Seventy-six percent (1,381) of the active wells were located in five refuges: Upper Ouachita and Delta NWRs in Louisiana, Kenai NWR in Alaska, and Hagerman and McFaddin NWRs in Texas.

The drive to increase oil and gas production necessitates a reexamination of the number of wells, seismic exploration activities, and pipelines affecting NWRS units. The goal of this study was to document the number of wells and pipelines on NWRS units and determine if major changes have occurred since the GAO [27] report. Objectives of this study were (1) to obtain current information on the number and status of wells and pipelines on NWRS units, (2) determine which NWRS units have the highest number of wells with no information on production type and status, and (3) recommend management actions to identify site-specific impacts, and strategies to remediate these impacts. Our assessment of oil and gas exploration and production on NWRS units should assist in the effective management of oil and gas activities on these lands.

## Methods

In order to evaluate the spatial coverage of oil and gas activities on NWRS units, we obtained data on well locations from the U.S. Environmental Protection Agency’s (EPA) Land-Based Oil and Gas Extraction database in June 2012 (EPA Database). We obtained data on pipelines crossing NWRS lands from the U.S. Department of Transportation Office of Pipeline Safety’s National Pipeline Mapping System in February 2012. The EPA data on wells was collected by HPDI Inc. (now known as DrillingInfo Inc., Austin, Texas) a private sector data gathering firm. In January 2011, DrillingInfo Inc. collected data on over 3 million wells from 30 state regulatory agencies or geological agencies and the Arizona Geological Society. This data included all oil and gas-producing states, with the exception of Illinois and Indiana. The EPA obtained well data from the Illinois State Geological Survey and the Indiana Geological Survey in February 2011.

**Fig 1:**
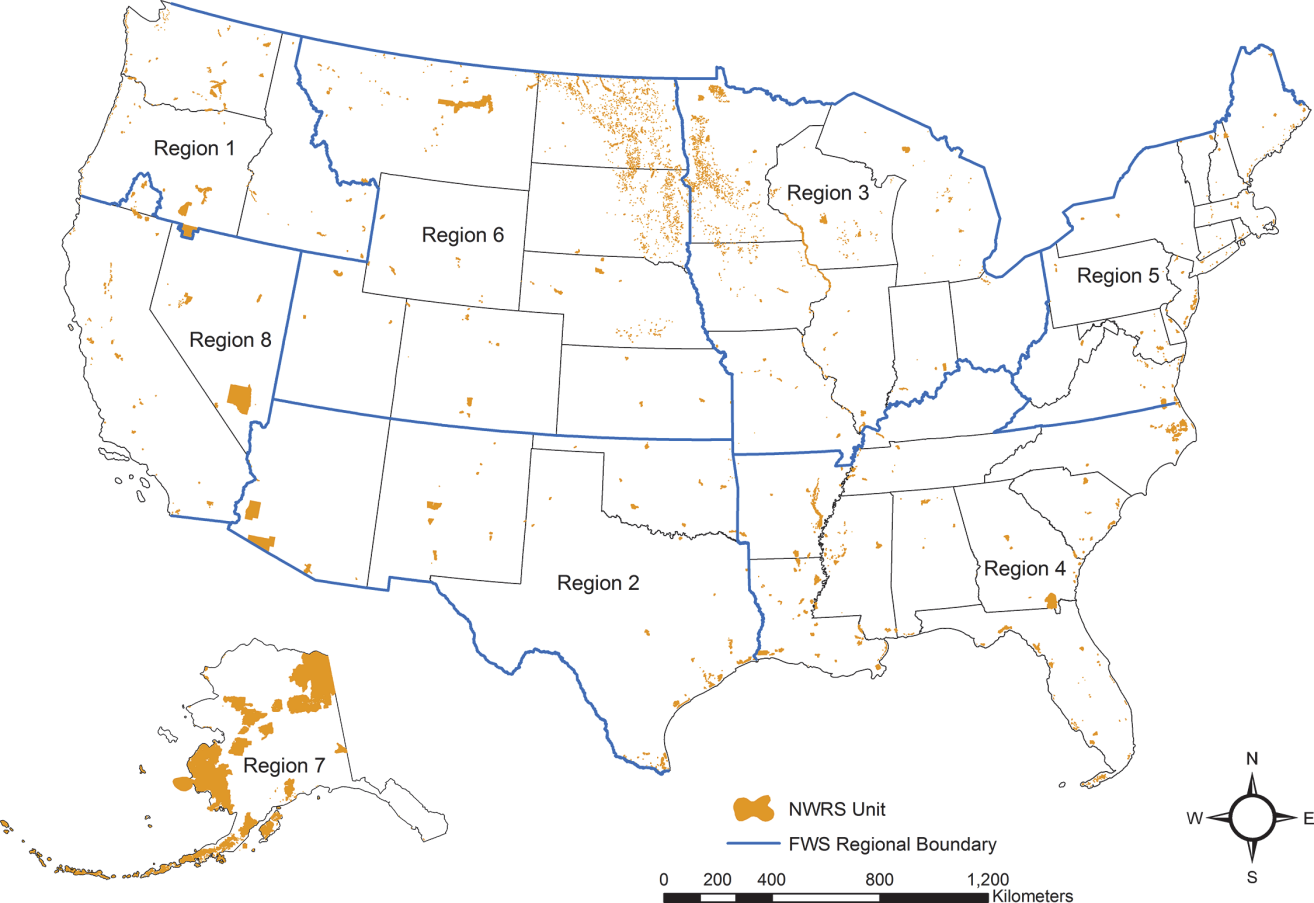
National Wildlife Refuges System Units in the United States. Locations of fee simple lands (*i*.*e*. absolute title to the surface land) in the U.S. Fish and Wildlife Service’s National Wildlife Refuge System (includes wildlife refuges, wildlife ranges, wildlife management areas, game preserves, conservation areas, and waterfowl production areas)(NWRS Units). U.S. Fish and Wildlife Service regional boundaries are depicted with blue lines (Pacific Islands in Region 1 and Puerto Rico in Region 4 excluded).

From these sources, we calculated the number of wells located within the NWRS. As of June 2012, this included 555 national wildlife refuges (NWRs), which include wildlife refuges, wildlife ranges, and wildlife management areas, 38 wetland management districts (WMD), and 206 waterfowl production areas (WPAs), covering over 60 million ha and located throughout the United States and its territories [26] (Fig 1). Analysis was restricted to NWRS lands owned in fee title (NWRS units) which included NWRs, WMDs, and WPAs. We also obtained data on well locations within 804 m outside of NWRS unit boundaries. We did not include conservation easements in our assessment.

The EPA Database categorizes the wells according to production type and status. Production type describes the fluid or gas produced or injected from or into the well (e.g., oil, gas, oil and gas, coal bed methane, carbon dioxide injection, water injection). Injection wells are used for the disposal of oilfield waste fluids and for the enhanced recovery of oil. Oilfield waste fluids such as saltwater or brine and wastewater from hydraulic fracturing operations are injected deep underground for disposal. Injection wells for waste fluid disposal are strictly regulated by the EPA and state regulatory agencies under Underground Injection Control regulations for the protection of aquifers. Injection wells are also used to inject water, steam, or carbon dioxide under pressure into the oil-producing formations to force more oil from the rock towards the producing wells. The status of a well relates to the current operation or condition of that facility (e.g., active, inactive, abandoned, dry hole, shut-in, plugged and abandoned, or unknown). “Active wells” are those that are actively producing, injecting, or disposing of fluids. “Inactive wells” are defined as unplugged wells that have had no reported production of oil and gas or both or injection for a period of a year or more. A “shut-in” well is one that is capable of production or injection and can be readily placed into production by activating existing equipment. “Plugged and abandoned” wells are plugged with cement and heavy mud and the wellhead removed. “Abandoned” wells include wells abandoned by oil operators due to lack of production or wells that have “failed beyond repair.” The EPA Database does not specify if wells designated as “abandoned” were plugged prior to abandonment. If the well did not produce oil or gas following well completion, the well was categorized as a dry hole, dry, or dry well.

We analyzed the spatial distribution of wells and pipelines across NWRS units using Geographic Information System (GIS) software (ArcGIS 10.1, ESRI, Redlands, CA). For the purposes of this study, we grouped wells into four general categories: gas, oil, oil and gas, and other. If the “production type” included gas individually or along with other types (except oil) such as injection well, enhanced oil recovery or saltwater disposal, we categorized the well as “gas.” Likewise, if the “production type” included oil and not gas we included the well in the “oil” category. If both oil and gas were included in the “production type,” we grouped the well as “oil and gas.” We categorized injection wells for enhanced oil recovery or wastewater disposal, coalbed methane wells, observation wells, stratigraphic wells, and water wells as “other.” We excluded wells classified as “cancelled” from the analysis as the permits for these “wells” were withdrawn by the operator and not drilled. We calculated the number of wells by production category and status within each NWRS unit area and aggregated individual NWRS unit counts to state and USFWS regional boundaries to examine regional trends. We visited the following NWRs in 2013 and 2014: Anahuac, McFaddin, and Hagerman in Texas; Deep Fork in Oklahoma; Delta, Atchafalaya, Catahoula, Lake Ophelia, and Tensas River in Louisiana; and St. Catherine Creek in Mississippi. We interviewed refuge managers and oil and gas specialists to identify issues with the oversight of oil and gas activities on refuges. Refuge managers and oil and gas specialists provided information on environmental effects of oil and gas exploration and production. We also obtained information on environmental compliance incidents from reports provided by refuge managers and oil and gas specialists. Using GIS, we measured the sizes of a subset of well pads from the following NWRs: Deep Fork (n = 14), Hagerman (n = 20), Tensas River (n = 15), Catahoula (n = 11), St. Catherine Creek (n = 13), Anahuac (n = 12), and McFaddin (n = 15). We selected no more than 10 well sites and 10 tank battery sites at each of the 7 NWRs.

## Results

### Wells on NWRS Units

**Fig 2:**
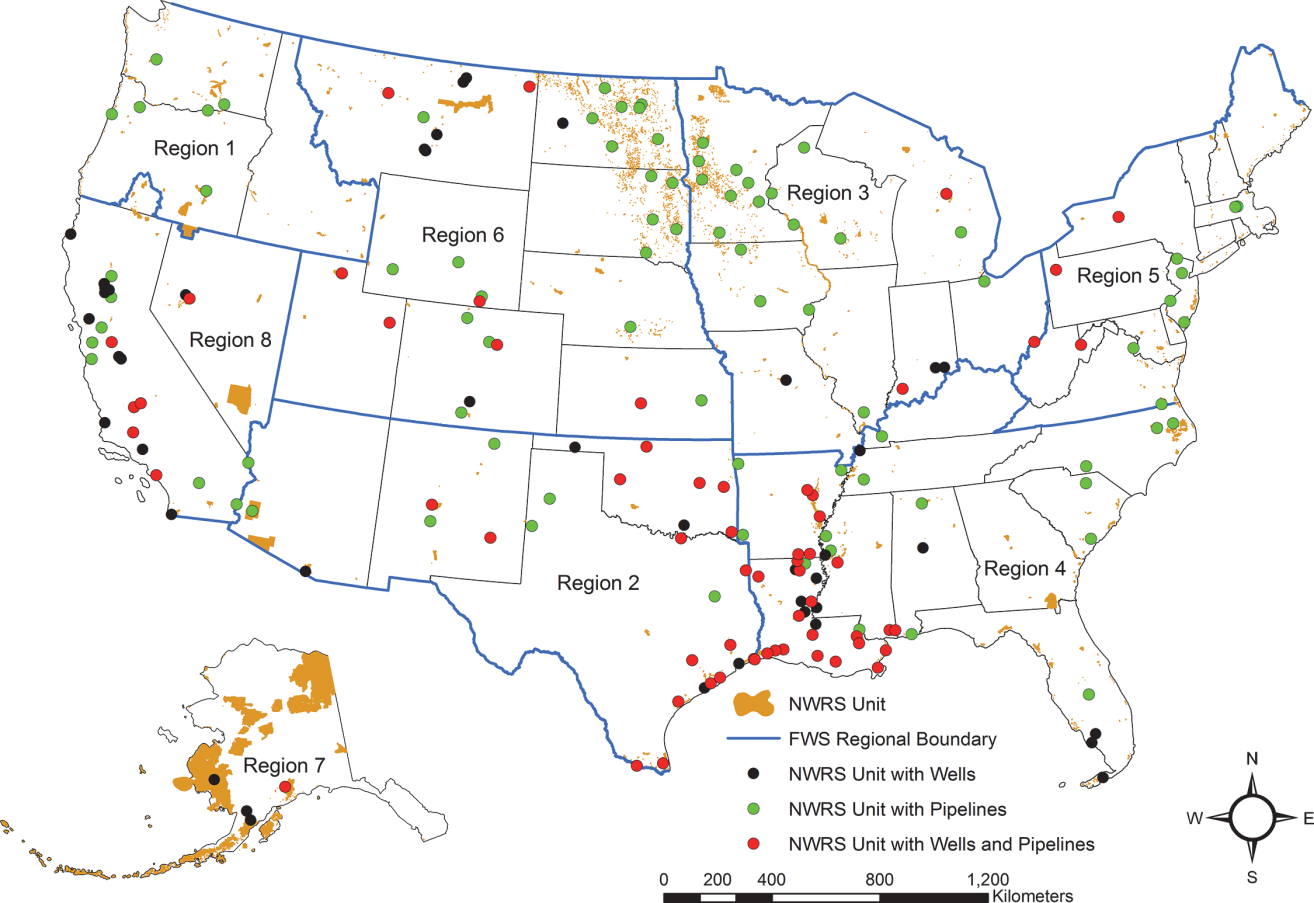
NWRS Units with Wells and Pipelines. Locations of National Wildlife Refuge System Units with oil and gas exploration and production wells and interstate pipelines. NWRS Units with no wells or pipelines (orange), NWRS Units with oil and gas exploration and production wells and no pipelines (black), NWRS Units with pipelines and no wells (green), and NWRS Units with wells and pipelines (red).

**Fig 3:**
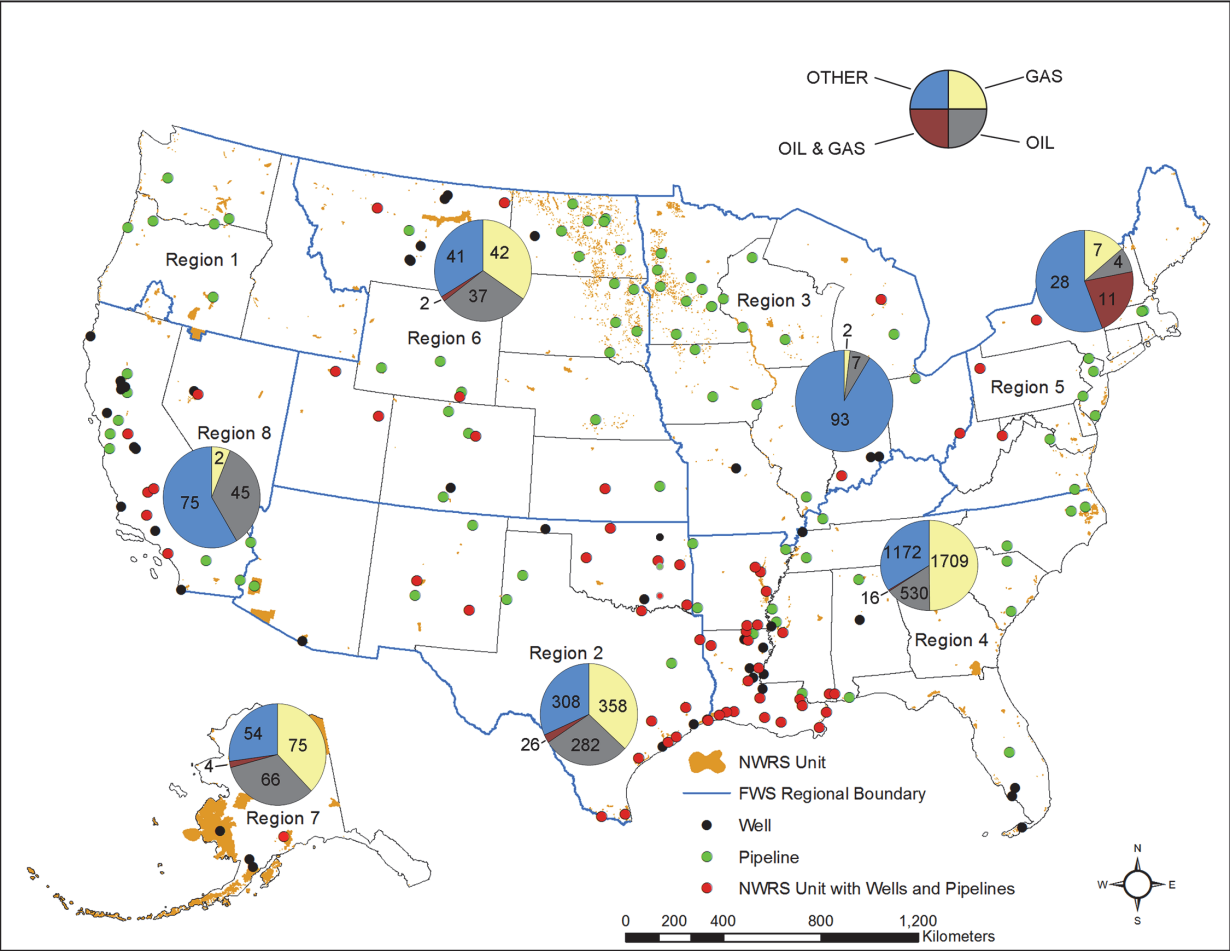
Number of Wells in NWRS Units by Region. Number and types of wells in National Wildlife Refuge System Units within U.S. Fish and Wildlife Service Regions. Pie charts show total number of wells by type for each region. Other wells include wells other than oil and gas, such as injection, saltwater disposal, enhanced oil recovery, dry, observation, stratigraphic, other, and production type data not available (N/A).

We identified 5,002 wells in 107 of the 599 NWRS units (Fig 2 and Table 1). The Southeast Region has the most NWRS units with wells and the most wells (35 NWRS units and 3,427 wells) followed by the Southwest Region (23 NWRS units and 974 wells) (Fig 3). Most of the wells in the Southeast and Southwest Regions are located in Louisiana, Mississippi, Oklahoma, and Texas. The Southwest, Southeast, and Mountain-Prairie Regions have the highest number of NWRS units with active wells 16, 14, and 9 NWRS units, respectively. The Southeast, Southwest, Pacific, and Mountain-Prairie Regions have the most NWRS units with inactive wells 35, 23, 18, and 17, respectively (Table 1). In terms of non-producing well sites, the Pacific Region has the most NWRS units with plugged and abandoned, dry, and shut-in wells (Table 2).

**Table 1:** Number of NWRS units (NWRs), listed by FWS region, with wells associated with oil and gas exploration and production within fee interest boundaries.

	Active Wells^1^			Inactive Wells^2^			All Wells		
FWS Region	Oil & Gas	Other^3^	Total NWRs	Oil & Gas	Other	Total NWRs	Oil & Gas	Other	Total NWRs
1 (Pacific)	0	0	0	0	0	0	0	0	0
2 (Southwest)	15	6	16	16	15	23	20	16	23
3 (Midwest)	2	2	2	1	4	5	2	4	5
4 (Southeast)	14	1	14	20	31	35	20	31	35
5 (Northeast)	0	0	0	3	3	4	3	3	4
6 (Mountain-Prairie)	9	0	9	8	14	17	11	14	18
7 (Alaska)	1	1	1	1	4	4	1	4	4
8 (Pacific Southwest)	3	1	3	6	18	18	6	18	18
Totals	44	11	45	55	89	106	63	90	107

^1^ Active wells—includes oil & gas wells that are producing oil and gas and other wells that are injecting gas or fluids underground

^2^ Inactive wells—includes all wells with a status ≠ active (e.g. inactive, plugged and abandoned, temporarily abandoned, shut-in, dry hole, unknown

^3^Other = includes wells other than oil and gas, such as injection, saltwater disposal, enhanced oil recovery, dry, observation, stratigraphic, other, and production type data not available (N/A).

**Table 2:** Number of NWRS units with non-producing oil and gas wells.

	Number of refuges by well status category				
FWS Region	Abandoned^1^	P & A^2^	Dry^3^	Shut-in^4^	N/A Inactive^5^
1 (Pacific)	0	0	0	0	0
2 (Southwest)	1	8	1	0	1
3 (Midwest)	1	0	3	0	1
4 (Southeast)	0	5	6	1	21
5 (Northeast)	0	2	3	0	0
6 (Mountain-Prairie)	0	3	0	1	10
7 (Alaska)	0	4	0	1	4
8 (Pacific Southwest)	0	16	16	4	0
Totals	2	38	29	7	37

^1^Abandoned—well no longer in use, whether dry, inoperable, or no longer productive, and where operators have intentionally relinquished interest in the well

^2^Plugged & Abandoned—well that has been plugged with cement & mud and has had wellhead removed

^3^Dry—well with no economically producible oil and/or gas

^4^Shut-in—a well capable of production or injection by opening valves or powering equipment

^5^N/A Inactive—well production type N/A (not available) and inactive status.

Thirty-three percent of the wells were categorized as “active,” while 44 percent of all wells were “inactive.” Only 14 percent of the wells were designated as plugged and abandoned (Table 3). Oil, gas or oil and gas combined make up 65 percent of all wells on NWRS units, with gas wells being the majority (Fig 3). Other wells include wells with production type listed as abandoned, injection, coalbed methane, observation, stratigraphic, dry hole, suspended, temporarily abandoned, unknown, or “blank” (*i*.*e*. no data). One percent (75 wells) of all wells within NWRS unit boundaries have other status codes including: application for permit to drill, drilling active, drilling suspended, dry, dry hole, other, suspended, temporarily abandoned, unknown, and “blank” or no data. Wells with no information on production type and inactive status made up 17 percent of all wells with almost all located in 21 NWRS units in the Southeast Region. Half of these wells were located in the following NWRs in Louisiana: Upper Ouachita (128 wells); D’Arbonne (100); Delta (86) and Tensas River (75). The number of abandoned wells in NWRS units is unknown as 318 wells did not have data on status (Status = N/A) and the EPA Database lists 2,196 wells as inactive. Inactive wells could include wells that are plugged and abandoned, shut-in, or unplugged and abandoned. Approximately 6,723 wells occur within 804 meters (m) (0.5 miles) outside of NWRS unit boundaries (Table 3). Twenty-eight percent (1,890 wells) of the wells located within 804 m of the NWRS unit boundaries are active wells and 41 percent (2,741 wells) are inactive. Thirteen percent of the wells (868) are plugged and abandoned, 11 percent (707) did not have data on status, six percent (430) were wells other than oil and gas, and one percent (87) were shut in. Twenty percent of the wells located within 804 m of the NWRS unit boundaries do not have data on production type (e.g. oil, gas, injection, etc.).

**Table 3:** Number of wells by Production Type and Status in and adjacent to NWRS units (On = within NWRS unit boundaries) (Adj = Adjacent within 804 meters (m) (0.5 miles) of NWRS unit boundaries).

	Gas		Oil		Oil & Gas		Other^4^		Total	
Well Status	On	Adj	On	Adj	On	Adj	On	Adj	On	Adj
Active	1282	977	257	419	8	399	118	95	1665	1890
Inactive	779	829	446	520	37	70	934	1322	2196	2741
P&A^1^	81	110	129	83	9	166	499	509	718	868
Shut-in	3	42	26	45	0	0	1	0	30	87
N/A^2^	53	107	106	177	3	65	156	358	318	707
Other Status^3^	3	41	7	20	2	218	63	151	75	430
Total	2201	2106	971	1264	59	918	1771	2435	5002	6723

^1^P&A = plugged and abandoned

^2^N/A = status data not available

^3^Other Status = includes other status codes not listed in table, such as dry hole, suspended, temporarily abandoned, unknown, or “blank” (*i*.*e*. no data)

^4^Other = includes wells other than oil and gas, such as injection, saltwater disposal, enhanced oil recovery, dry, observation, stratigraphic, other, and production type data not available (N/A).

Injection wells make up five percent of all wells on NWRS units, with over half designated as active (Table 4). The Southwest Region has the most NWRS units (11) with injection wells. Deep Fork and Hagerman NWRs have the highest number of injection wells in the NWRS, 82 and 68, respectively. Almost all of the injection wells at Deep Fork NWR are active. Of the 21 active injection wells at Hagerman NWR, one is used for saltwater (oilfield brine) disposal and the remainder are used for secondary recovery (waterflood) of oil. Five NWRS units contain 65 percent of all oil and gas wells (excluding other well types such as injection wells) in the NWRS with three of the top five NWRS units located in Louisiana (Table 5). Deep Fork NWR has the highest number of active oil wells and Upper Ouachita NWR has the highest number of active gas wells in the NWRS.

**Table 4:** Number of injection wells on NWRS units.

	Injection well status					
	Active	Inactive	P & A	Other Status^1^	Total	Number of NWRS units
1 (Pacific)	0	0	0	0	0	0
2 (Southwest)	112	21	15	13	161	11
3 (Midwest)	1	4	0	0	5	1
4 (Southeast)	5	1	20	7	33	2
5 (Northeast)	0	0	0	0	0	0
6 (Mountain-Prairie)	0	2	0	0	2	1
7 (Alaska)	2	0	0	0	2	1
8 (Pacific Southwest)	1	1	0	1	3	1
Totals	121	29	35	21	206	17

^1^ Other Status—temporarily abandoned, drilling, well status not available, or shut-in.

**Table 5:** NWRS units with the highest number of oil and gas wells (excluding other wells such as injection wells).

		Number of Wells						
NWRS unit	State	Oil		Gas		Oil & Gas		Total
		Active	Inactive	Active	Inactive	Active	Inactive	All Wells
Upper Ouachita NWR	LA	0	2	928	352	0	0	1,282
Delta NWR	LA	18	196	3	49	0	11	277
D’Arbonne NWR	LA	0	0	100	83	0	0	183
Deep Fork NWR	OK	68	79	2	24	0	0	173
Lower Rio Grande Valley NWR	TX	4	19	60	79	2	8	172
	Totals	90	296	1,093	587	2	19	2,087

### Pipelines on NWRS Fee-Interest Lands

There are 595 pipelines totaling approximately 2,155 km (1,339 miles) that cross 173 NWRS units (Table 6 and Fig 2). Nineteen percent of the 599 NWRS units included in the analysis only have pipelines crossing the NWRS units and no wells associated with oil and gas production. Pipeline construction on some NWRS units occurred before the USFWS acquired the property. The Southeast Region has the most NWRS units with pipelines followed by the Mountain-Prairie Region (Table 6). Sixty-eight percent of the pipelines traversing NWRS units transport gas with most of these pipelines occurring in the Southwest and Southeast Regions. Almost all of the gas pipelines (96 percent) transport natural gas and the remainder convey carbon dioxide, hydrogen or other gases. Liquids pipelines transport crude oil, anhydrous ammonia, liquefied petroleum gas, natural gas liquids, highly volatile liquids, and non-highly volatile liquid products. Most pipelines are from five to 106 centimeters in diameter and are buried underground.

**Table 6:** Number of pipelines crossing NWRS units.

	Number of Pipelines					
FWS Region	Liquids^1^	Gas^2^	Total	Km of Pipeline	Number of NWRS units with Pipelines	Number of NWRS units with Pipelines Only^3^
1 (Pacific)	3	5	8	42	6	6
2 (Southwest)	76	106	182	745	26	8
3 (Midwest)	28	70	98	237	21	19
4 (Southeast)	34	156	190	724	43	19
5 (Northeast)	4	21	25	53	12	8
6 (Mountain-Prairie)	23	37	60	158	28	21
7 (Alaska)	2	5	7	163	1	0
8 (Pacific Southwest)	1	24	25	32	12	6
Totals	171	424	595	2,153	149	87

^1^ Liquids pipelines transport crude oil, and refined products such as gasoline, diesel, and jet fuel.

^2^ Gas pipelines transport natural gas and other gases such as carbon dioxide.

^3^ NWRS units having pipelines only and no oil and gas wells.

### Environmental Effects of Oil and Gas on NWRS Units

During our visits to refuges in Louisiana, Oklahoma, and Texas we identified the following issues related to oil and gas activities: chronic oil and brine leaks and spills at oil production sites, dead vegetation due to oilfield brine spills, trash, and abandoned wells and oilfield equipment. We documented abandoned oilfield equipment including pump jacks, pipes, separators, flowlines, and storage tanks in the following NWRs: Atchafalaya, Delta, and Tensas River in Louisiana; Deep Fork in Oklahoma; and Anahuac, Brazoria, Hagerman, McFaddin, and San Bernard in Texas. Well pad sizes ranged from 0.01 to 1 ha. Well sites averaged 0.4 ha and well pads with tank batteries generally were larger (average 1 ha). NWRS unit managers and oil and gas specialists reported improvement in working with oil and gas operators to achieve resource protection; however, they added that unnecessary impacts on NWRS units still occur.

## Discussion

Even though NWRS units are managed “to conserve, protect, and enhance fish, wildlife, plants, and their habitats for the continuing benefit of the American people” (USFWS Mission Statement), these public lands vary from pristine, relatively undisturbed habitats to areas degraded by land uses, such as oil and gas and agriculture, occurring prior the acquisition of these lands for conservation [28]. Some oil and gas wells in Deep Fork NWR in Oklahoma and Upper Ouachita NWR in Louisiana were drilled during the 1920s and 1930s, over 50 years prior to the establishment of these NWRs.

The Southeast Region continues to have the most NWRS units with wells and over half of the wells in the NWRS. Twenty-five percent of the wells in the NWRS are located at Upper Ouachita NWR in north-central Louisiana. All but two of the wells at this NWR are gas wells and over half of the wells were completed prior to the acquisition of the NWR in 1978. Deregulation of natural gas prices in the 1970s led to an increase in drilling at Upper Ouachita NWR with some wells drilled with spacing of 183 meters (600 feet) or less, hence the large number of wells [29]. Wells at the Upper Ouachita NWR produce natural gas from the Monroe gas field, Louisiana’s largest at approximately 945 km^2^ [30]. D’Arbonne and Black Bayou Lake NWRs are also located in the Monroe Gas Field and rank second and third, respectively, in the number of active gas wells.

Since publication of the GAO findings in 2003 [27], the NWRS increased from 575 to 599 NWRS units. During the same period, the number of wells increased from 4,406 to 5,002 wells. We could not attribute the increase in wells to an increase in the number of NWRS units alone as none of the 24 NWRS units acquired since 2003 have any wells within their boundaries. The increase may be due to the acquisition of additional land for existing NWRS units or the drilling of additional wells. We could not directly compare the well numbers reported by the GAO [27] to our data, as we did not have specific information on the actual NWRS unit fee simple boundaries used in the GAO analysis or information on how the GAO imported the well data from the data sources. There was an eight percent decline in active wells from 1,806 wells in 2003 to 1,665 wells in 2011 probably due to a decline in oil and gas production as the wells aged. Well status can change from active to shut-in, abandoned, or plugged and abandoned as oil and gas production in the well declines. The numbers of plugged and abandoned and inactive wells did not vary considerably between 2003 and 2011. In 2003, 57 percent of the wells were located in the following five NWRs: the Upper Ouachita and Delta in Louisiana, St. Catherine Creek in Mississippi, Deep Fork in Oklahoma, and the Lower Rio Grande Valley in Texas [27]. Although St. Catherine Creek ranked as one of the five NWRs with the highest number of wells in 2003, 90 percent of the wells were plugged and abandoned after 2003.

The number of injection wells doubled from 2003 to 2011 although injection wells comprised less than 5 percent of the total number of wells in the NWRS. As the wells age, oil and gas production typically declines and the volume of produced water increases; this may account for the increase in injection wells for wastewater disposal or enhanced oil recovery. Oil operators typically convert dry holes or unproductive oil and gas wells into injection wells to enhance the recovery of oil or for produced water disposal.

Active wells pose a threat to NWRS units due to spills and disturbance from routine operation and maintenance. Inactive wells present environmental and economic risks. Although inactive wells are not producing or injecting fluids, there is still a risk of leakage onto the surface or contamination of aquifers due to the loss of wellbore integrity. Delays in the plugging and abandonment of inactive wells increase the risk of well casing and cement failure. Additionally, oil operators can abandon inactive wells due to inadequate finances passing the cost of plugging and abandonment as well as site reclamation to the taxpayer.

Unplugged wells and improperly plugged wells can provide a pathway for oil and other hydrocarbons, well stimulation chemicals, and oilfield brine to aquifers or to the surface and contaminate ground water, water wells, soils, vegetation, and surface waters. In addition to the environmental risks, unplugged and improperly plugged wells pose a risk to public safety as surface seeps can increase the threat and intensity of wildfires [31]. In 2011, three orphan wells posing the risk of contamination to a lake at the Lower Rio Grande Valley NWR were plugged and abandoned at a cost of over $1 million [32]. An oil well plugged and abandoned in 1983 at St. Catherine Creek NWR leaked in April 2012 requiring replugging of the well and site restoration at a cost exceeding $260,000 (M Cupit, U.S. Fish and Wildlife Service, pers. comm.).

The Interstate Oil and Gas Compact Commission (IOGCC) states that “*many wells pre-dating 1952 were probably plugged improperly”* because cement plugs from that time period “*were not always effective as their compounds lacked the chemical components to withstand down-hole temperatures and pressure*” and thus, probably failed to harden and seal the wells properly [31]. The IOGCC [31] estimates that over 90,000 orphan wells exist in the United States with 60,000 wells on waiting lists for plugging by State oil and gas agencies. The IOGCC defines an orphan well as “*a well that is not producing or injecting*, *has not received state approval to remain idle*, *and for which the operator is unknown or insolvent*.” The GAO [27] did not report the number of abandoned wells located in NWRS units; however, the GAO [27] reported 59 percent of the wells as “inactive with an unknown number of these wells abandoned but not plugged.” The number of unplugged and abandoned wells as well as orphan wells in NWRS units remains unknown, as is the number of well sites in need of restoration. With well sites averaging in size from 0.4 to 1 ha, the number of sites requiring restoration could be significant. Surface equipment abandoned along with wells can pose a hazard to refuges and public safety. Above ground flowlines and well casings protruding above the ground surface present a safety risk to refuge personnel engaged in management activities such as mowing. Dense vegetation can hide these pipes, which can damage refuge equipment and vehicles. Oilfield equipment abandoned on a refuge can restrict a refuge manager’s restoration and management options.

Spills on NWRS units ranged from small chronic oil spills within a well production site to large spills involving flowline leaks releasing large volumes of brine into the environment in NWRS units. In March 2013, a drilling operation spilled 25 bbls of oil-based drilling mud at the Lower Rio Grande Valley NWR. The drilling mud spill resulted in the contamination of soil and vegetation adjacent to the well pad (M Maddux, U.S. Fish and Wildlife Service, pers. comm.). Brine spills present longer-term impacts to NWRS units than oil spills as remediation or recovery of brine spill sites is slower due to loss of vegetation and damage to soils [33]. In 2010, Hagerman NWR reported six brine spills primarily due to flowline leaks. Corrosion of a flowline caused the release of 75 bbls of oilfield brine at Hagerman NWR in February 29, 2012. This spill affected 0.8 ha of habitat, killed 84 hardwood trees over 150-years old, and caused an estimated $154,000 in damages (M Maddux, U.S. Fish and Wildlife Service, pers. comm.). A review of brine and oil spill data from Oklahoma [14] and North Dakota [34] shows that the rate of spill incidents ranges from one spill for every 53 to76 active wells. Fisher and Sublette [14] found the average size of spills at oil production sites in Oklahoma ranged from 34 to 46 bbls of oil and 89 to 158 bbls of brine per spill. On average, oil production facilities resulted in the release of 62,000 bbls of oil and 146,000 bbls of brine. Fisher and Sublette [14] determined that brine and crude oil comprised 76 percent of all fluid released from oil production facilities with 34 percent of the spills resulting in injury to surface water, crops, livestock, soil, fish or wildlife. Even though not all spills at oil production facilities result in injury to natural resources, those in NWRS units are a concern given the sensitive nature of these protected areas. Although states like Oklahoma and North Dakota maintain databases on the number of spills, not all states track the smaller oil and brine spills unless they impact surface waters.

Oil and gas production facilities as well as well completion operations can release methane and volatile organic compounds (VOCs) such as benzene, toluene, ethylbenzene, and xylenes (BTEX) into the atmosphere [35, 36, 37]. Emission sources at oil and gas production facilities include glycol dehydrators, pumps, and condensate tanks. Condensates are very light liquid hydrocarbons that condense out of natural gas at the surface and are referred to as natural gasoline [38]. VOC emissions from condensate occur when the liquid hydrocarbon is brought to the surface where the decreased atmospheric pressure causes gases dissolved in the condensate to volatilize [36]. VOC emissions also occur during drilling, hydraulic fracturing, and well restimulation or maintenance [36]. The impacts of these emissions to refuge resources are unknown.

Advances in oil and gas drilling and production technology have made previously uneconomical oil and gas-bearing formations, such as shale oil and shale gas economically viable to extract. Consequently, the development of shale oil and shale gas formations, in particular the Bakken in North Dakota and Eastern Montana and the Marcellus in Ohio, West Virginia, Pennsylvania, and New York could affect NWRS lands in these states. Oil and gas exploration and drilling in prospective shale formations like the Tuscaloosa marine shale in central Louisiana could potentially increase the amount of oil and gas activity in the following NWRs: Bogue Chitto, Cat Island, Catahoula, Grand Cote, Lake Ophelia, and St. Catherine Creek.

Environmental impacts from oil and gas pipelines on NWRS units include spills resulting in wildlife mortality, and soil and sediment contamination [27] as well as habitat alternation from the construction and maintenance of pipeline rights-of-way. Pipeline rights-of-way provide access along the pipeline route for inspection and maintenance. The rights-of-way also provide access for unauthorized uses such as poaching or off-road motorized vehicles (P. O’Dell, U.S. Fish and Wildlife Service, pers. comm.). Land disturbance for pipeline construction and maintenance can also lead to the introduction of invasive species. Pipeline spills can cause significant damage to terrestrial and aquatic habitats. The leading causes of releases from pipelines include: punctures or other damage from excavators and agricultural activities; corrosion, manufacturing defects in the pipe, construction flaws in pipe installation or welding, natural hazards such as ground movement, weather, lightning, and stream currents [39]. Pipelines buried under rivers and streams can be exposed by erosion and become susceptible to damage or rupture by strong currents [39].

## Conclusions and Management Recommendations

The updated data on oil and gas wells and pipelines in and adjacent to NWRS lands will assist the USFWS in determining follow-up assessments on the status of these wells and impacts to individual NWRS units. Although the updated data are useful, errors in the collection of data on thousands of wells are inherent. Given the data uncertainty, follow-up assessments on oil and gas wells should be conducted to verify well status by contacting the pertinent state oil and gas regulatory agencies. Protocols should be developed for the follow-up inventory of oil and gas wells and for monitoring these sites. This will provide consistency for data collection on well locations, types, and status as well as information on oil and gas production site spills, impacts to refuge resources, and environmental compliance.

On-site ecological assessments should be conducted on NWRS units with oil and gas activities to determine impacts from brine, oil and other hydrocarbon spills, as well as habitat alteration, and other impacts associated with oil and gas extraction. The on-site ecological assessments should also identify abandoned oil and gas facilities needing equipment removal and site restoration. Several states have programs to fund these activities. The on-site ecological assessments should determine the nature and extent of contamination, the source, and cause of spills and releases, injury to NWRS unit resources, and corrective measures to prevent continued or future harm to the NWRS unit. Land managers should forward information on abandoned oil and gas facilities requiring removal and site restoration to the appropriate state regulatory agencies and oil operators and request removal of equipment and site restoration.

Oil operators should develop operation and maintenance plans for existing oil and gas production facilities in NWRS units. The operation and maintenance plans should include, at a minimum, spill prevention, control and countermeasures (SPCC); waste management and disposal; procedures for reporting spills or releases to the land manager; and procedures for routine maintenance of the facilities and for maintenance of the well using workover rigs or other heavy equipment. Spill contingency plans should be developed for NWRS units with pipelines or for NWRS units with pipelines crossing waterways upstream of NWRS unit boundaries.

Oil and gas activities on NWR lands are not generally aligned with the NWRS mission to “administer a national network of lands and waters for the conservation, management, and where appropriate, restoration of the fish, wildlife, and plant resources and their habitats within the United States for the benefit of present and future generations of Americans.” While the USFWS has had many local successes working with oil and gas operators to achieve appropriate resource protections, there are many more examples of unnecessary impacts on trust resources and refuge management. The USFWS has made progress in training NWRS staff in the oversight of oil and gas activities with the goal of avoiding or minimizing impacts to refuge resources. However, given the increase in oil and gas development, a strong, comprehensive and cohesive oil and gas management program across the USFWS is essential to a proper balance between the NWRS mission and the reasonable exercise of private oil and gas rights. Such a management program needs, at its core, a consolidated and robust regulatory framework as recommended by the GAO [27] to “*strengthen and provide greater consistency*” in the management and oversight of oil and gas activities on refuges and “*to protect the public’s surface interests*.*”*.

## Supporting Information

S1 Table Number of oil and gas exploration and production wells and interstate pipelines on National Wildlife Refuge System fee simple lands (i.e. absolute title to the surface land). (DOCX)

S2 Table Injection wells on NWRS units. (DOCX)

S3 Table Number of wells in NWRS units by Status. [P&A = plugged and abandoned; N/A = status data not available; Other Status = includes other status codes not listed in table, such as dry hole, suspended, temporarily abandoned, unknown, or “blank” (i.e. not data)].
(DOCX)

S4 Table Number of wells in NWRS units by production type and status. (DOCX)
