# Supplementary material for: Oil and Gas Wells and Pipelines on U.S. Wildlife Refuges: Challenges for Managers
Source: PLoS One. 2015 Apr 27;10(4):e0124085. doi: 10.1371/journal.pone.0124085 (PMC4410920; doi:10.1371/journal.pone.0124085)
Supplement: S1 Table — (DOCX) [file pone.0124085.s001.docx]

| **Region / State / NWRS Unit** | **Gas** | **Oil** | **Oil and Gas** | **Other** | **Total** | **Pipelines** |
| --- | --- | --- | --- | --- | --- | --- |
| **Northwest Region (1)** | **0** | **0** | **0** | **0** | **0** | **8** |
| ***Oregon*** |  |  |  |  |  | **4** |
| Malheur National Wildlife Refuge |  |  |  |  |  | 1 |
| McNary National Wildlife Refuge |  |  |  |  |  | 1 |
| Siletz Bay National Wildlife Refuge |  |  |  |  |  | 1 |
| Tualatin River National Wildlife Refuge |  |  |  |  |  | 1 |
| ***Washington*** |  |  |  |  |  | **4** |
| McNary National Wildlife Refuge |  |  |  |  |  | 2 |
| Nisqually National Wildlife Refuge |  |  |  |  |  | 1 |
| Umatilla National Wildlife Refuge |  |  |  |  |  | 1 |
|  |  |  |  |  |  |  |
| **Southwest Region (2)** | **358** | **282** | **26** | **308** | **974** | **182** |
| ***Arizona*** |  |  |  | **1** | **1** | **14** |
| Buenos Aires National Wildlife Refuge |  |  |  | 1 | 1 |  |
| Havasu National Wildlife Refuge |  |  |  |  |  | 10 |
| Kofa National Wildlife Refuge |  |  |  |  |  | 4 |
| ***New Mexico*** | **5** | **10** |  | **2** | **17** | **6** |
| Bitter Lake National Wildlife Refuge | 5 | 10 |  |  | 15 | 2 |
| Bosque Del Apache National Wildlife Refuge |  |  |  |  |  | 1 |
| Maxwell National Wildlife Refuge |  |  |  |  |  | 1 |
| Sevilleta National Wildlife Refuge |  |  |  | 2 | 2 | 2 |
| ***Oklahoma*** | **62** | **149** |  | **255** | **466** | **9** |
| Deep Fork National Wildlife Refuge | 26 | 147 |  | 227 | 400 | 4 |
| Little River National Wildlife Refuge |  | 1 |  | 7 | 8 | 1 |
| Optima National Wildlife Refuge | 13 |  |  | 2 | 15 |  |
| Salt Plains National Wildlife Refuge | 11 | 1 |  | 1 | 13 | 2 |
| Sequoyah National Wildlife Refuge |  |  |  | 4 | 4 | 1 |
| Tishomingo National Wildlife Refuge | 2 |  |  | 12 | 14 |  |
| Washita National Wildlife Refuge | 10 |  |  | 2 | 12 | 1 |
| ***Texas*** | **291** | **123** | **26** | **50** | **490** | **153** |
| Anahuac National Wildlife Refuge | 5 | 7 |  | 3 | 15 |  |
| Aransas National Wildlife Refuge | 58 | 8 | 4 | 2 | 72 | 2 |
| Attwater Prairie Chicken National Wildlife Refuge | 19 | 1 |  |  | 20 | 5 |
| Big Boggy National Wildlife Refuge | 1 |  |  |  | 1 |  |
| Brazoria National Wildlife Refuge | 12 | 4 | 3 |  | 19 | 29 |
| Buffalo Lake National Wildlife Refuge |  |  |  |  |  | 1 |
| Caddo Lake National Wildlife Refuge | 2 | 2 |  | 1 | 5 | 2 |
| Hagerman National Wildlife Refuge | 3 | 71 | 4 | 38 | 116 | 1 |
| Laguna Atascosa National Wildlife Refuge | 11 |  |  |  | 11 | 1 |
| Lower Rio Grande Valley National Wildlife Refuge | 139 | 23 | 10 | 3 | 175 | 23 |
| **Region / State / NWRS Unit** | **Gas** | **Oil** | **Oil and Gas** | **Other** | **Total** | **Pipelines** |
| McFaddin National Wildlife Refuge | 14 | 4 |  | 2 | 20 | 3 |
| Muleshoe National Wildlife Refuge |  |  |  |  |  | 1 |
| Neches River National Wildlife Refuge |  |  |  |  |  | 3 |
| San Bernard National Wildlife Refuge | 24 | 3 | 2 | 1 | 30 | 58 |
| Texas Point National Wildlife Refuge |  |  | 2 |  | 2 | 4 |
| Trinity River National Wildlife Refuge | 3 |  | 1 |  | 4 | 20 |
|  |  |  |  |  |  |  |
| **Midwest Region (3)** | **2** | **7** |  | **93** | **102** | **98** |
| ***Illinois*** |  |  |  |  |  | **8** |
| Crab Orchard National Wildlife Refuge |  |  |  |  |  | 2 |
| Port Louisa National Wildlife Refuge |  |  |  |  |  | 2 |
| Upper Mississippi River National Wildlife And Fish Refuge |  |  |  |  |  | 4 |
| ***Indiana*** |  | **7** |  | **89** | **96** | **7** |
| Big Oaks National Wildlife Refuge |  |  |  | 5 | 5 |  |
| Muscatatuck National Wildlife Refuge |  |  |  | 1 | 1 |  |
| Patoka River National Wildlife Refuge |  | 7 |  | 83 | 90 | 7 |
| ***Iowa*** |  |  |  |  |  | ***20*** |
| Iowa Wetland Management District |  |  |  |  |  | 14 |
| Neal Smith National Wildlife Refuge |  |  |  |  |  | 1 |
| Port Louisa National Wildlife Refuge |  |  |  |  |  | 2 |
| Upper Mississippi River National Wildlife And Fish Refuge |  |  |  |  |  | 3 |
| ***Michigan*** | **2** |  |  |  | **2** | **18** |
| Kirtlands Warbler Wildlife Management Area | 2 |  |  |  | 2 | 17 |
| Shiawassee National Wildlife Refuge |  |  |  |  |  | 1 |
| ***Minnesota*** |  |  |  |  |  | **33** |
| Crane Meadows National Wildlife Refuge |  |  |  |  |  | 1 |
| Detroit Lakes Wetland Management District |  |  |  |  |  | 1 |
| Fergus Falls Wetland Management District |  |  |  |  |  | 7 |
| Litchfield Wetland Management District |  |  |  |  |  | 2 |
| Minnesota Valley National Wildlife Refuge |  |  |  |  |  | 5 |
| Minnesota Valley Wetland Management District |  |  |  |  |  | 2 |
| Morris Wetland Management District |  |  |  |  |  | 5 |
| Sherburne National Wildlife Refuge |  |  |  |  |  | 1 |
| Upper Mississippi River National Wildlife And Fish Refuge |  |  |  |  |  | 1 |
| Windom Wetland Management District |  |  |  |  |  | 8 |
| ***Missouri*** |  |  |  | **4** | **4** |  |
| Big Muddy National Fish And Wildlife Refuge |  |  |  | 4 | 4 |  |
| ***Ohio*** |  |  |  |  |  | **1** |
| Ottawa National Wildlife Refuge |  |  |  |  |  | 1 |
|  |  |  |  |  |  |  |
| **Region / State / NWRS Unit** | **Gas** | **Oil** | **Oil and Gas** | **Other** | **Total** | **Pipelines** |
| ***Wisconsin*** |  |  |  |  |  | **11** |
| Leopold Wetland Management District |  |  |  |  |  | 7 |
| St. Croix Wetland Management District |  |  |  |  |  | 2 |
| Upper Mississippi River National Wildlife And Fish Refuge |  |  |  |  |  | 1 |
| Whittlesey Creek National Wildlife Refuge |  |  |  |  |  | 1 |
|  |  |  |  |  |  |  |
| **Southeast Region (4)** | **1709** | **530** | **16** | **1172** | **3427** | **190** |
| ***Alabama*** |  |  |  | **14** | **14** | **4** |
| Cahaba River National Wildlife Refuge |  |  |  | 14 | 14 |  |
| Grand Bay National Wildlife Refuge |  |  |  |  |  | 2 |
| Wheeler National Wildlife Refuge |  |  |  |  |  | 2 |
| ***Arkansas*** | **8** | **51** |  | **6** | **65** | **25** |
| Bald Knob National Wildlife Refuge | 3 |  |  |  | 3 | 13 |
| Big Lake National Wildlife Refuge |  |  |  |  |  | 1 |
| Cache River National Wildlife Refuge | 2 |  |  |  | 2 | 3 |
| Felsenthal National Wildlife Refuge | 3 | 48 |  | 6 | 57 | 1 |
| Logan Cave National Wildlife Refuge |  |  |  |  |  | 2 |
| Overflow National Wildlife Refuge |  | 1 |  |  | 1 | 1 |
| Pond Creek National Wildlife Refuge |  |  |  |  |  | 1 |
| White River National Wildlife Refuge |  | 2 |  |  | 2 | 3 |
| ***Florida*** |  |  |  | **4** | **4** | **1** |
| Florida Panther National Wildlife Refuge |  |  |  | 2 | 2 |  |
| Lake Wales Ridge National Wildlife Refuge |  |  |  |  |  | 1 |
| National Key Deer Refuge |  |  |  | 1 | 1 |  |
| Ten Thousand Islands National Wildlife Refuge |  |  |  | 1 | 1 |  |
| ***Georgia*** |  |  |  |  |  | **1** |
| Savannah National Wildlife Refuge |  |  |  |  |  | 1 |
| ***Kentucky*** |  |  |  | **1** | **1** | **3** |
| Clarks River National Wildlife Refuge |  |  |  |  |  | 3 |
| Reelfoot National Wildlife Refuge |  |  |  | 1 | 1 |  |
| ***Louisiana*** | **1693** | **349** | **16** | **772** | **2830** | **130** |
| Atchafalaya National Wildlife Refuge | 9 | 12 | 2 | 23 | 46 | 8 |
| Bayou Cocodrie National Wildlife Refuge |  |  |  | 49 | 49 | 1 |
| Bayou Sauvage National Wildlife Refuge |  |  |  | 4 | 4 | 10 |
| Bayou Teche National Wildlife Refuge | 14 | 3 |  | 18 | 35 | 26 |
| Big Branch Marsh National Wildlife Refuge |  |  |  | 4 | 4 | 5 |
| Black Bayou Lake National Wildlife Refuge | 78 |  |  | 7 | 85 | 7 |
| Bogue Chitto National Wildlife Refuge |  |  |  |  |  | 1 |
| Breton National Wildlife Refuge |  |  |  | 3 | 3 | 2 |
| Cameron Prairie National Wildlife Refuge |  |  |  | 14 | 14 | 3 |
| **Region / State / NWRS Unit** | **Gas** | **Oil** | **Oil and Gas** | **Other** | **Total** | **Pipelines** |
| Cat Island National Wildlife Refuge | 2 |  |  | 3 | 5 |  |
| Catahoula National Wildlife Refuge | 1 | 20 |  | 54 | 75 |  |
| D'Arbonne National Wildlife Refuge | 183 |  |  | 100 | 283 | 8 |
| Delta National Wildlife Refuge | 52 | 214 | 11 | 86 | 363 | 14 |
| Grand Cote National Wildlife Refuge |  |  |  | 2 | 2 | 1 |
| Handy Brake National Wildlife Refuge |  |  |  |  |  | 6 |
| Lacassine National Wildlife Refuge | 32 | 5 | 1 | 42 | 80 | 14 |
| Lake Ophelia National Wildlife Refuge |  | 5 |  | 51 | 56 |  |
| Mandalay National Wildlife Refuge | 13 | 5 |  | 25 | 43 | 4 |
| Red River National Wildlife Refuge | 6 | 24 |  | 27 | 57 | 8 |
| Sabine National Wildlife Refuge | 22 | 23 | 2 | 51 | 98 | 10 |
| St. Catherine Creek National Wildlife Refuge |  |  |  | 6 | 6 |  |
| Tensas River National Wildlife Refuge | 1 | 36 |  | 75 | 112 |  |
| Upper Ouachita National Wildlife Refuge | 1280 | 2 |  | 128 | 1410 | 2 |
| ***Mississippi*** | **8** | **130** |  | **375** | **513** | **16** |
| Dahomey National Wildlife Refuge |  |  |  |  |  | 4 |
| Grand Bay National Wildlife Refuge |  |  |  | 1 | 1 | 2 |
| Holt Collier National Wildlife Refuge |  |  |  |  |  | 1 |
| Mississippi Sandhill Crane National Wildlife Refuge |  |  |  | 1 | 1 | 2 |
| Panther Swamp National Wildlife Refuge |  |  |  | 2 | 2 | 4 |
| St. Catherine Creek National Wildlife Refuge | 8 | 130 |  | 370 | 508 |  |
| Sam D. Hamilton Noxubee National Wildlife Refuge |  |  |  |  |  | 3 |
| Yazoo National Wildlife Refuge |  |  |  | 1 | 1 |  |
| ***North Carolina*** |  |  |  |  |  | **6** |
| Pee Dee National Wildlife Refuge |  |  |  |  |  | 3 |
| Pocosin Lakes National Wildlife Refuge |  |  |  |  |  | 2 |
| Roanoke River National Wildlife Refuge |  |  |  |  |  | 1 |
| ***South Carolina*** |  |  |  |  |  | **3** |
| Carolina Sandhills National Wildlife Refuge |  |  |  |  |  | 1 |
| Ernest F. Hollings Ace Basin National Wildlife Refuge |  |  |  |  |  | 1 |
| Savannah National Wildlife Refuge |  |  |  |  |  | 1 |
| ***Tennessee*** |  |  |  |  |  | **1** |
| Hatchie National Wildlife Refuge |  |  |  |  |  | 1 |
|  |  |  |  |  |  |  |
| **Northeast Region (5)** | **7** | **4** | **11** | **28** | **50** | **25** |
| ***Massachusetts*** |  |  |  |  |  | **2** |
| Assabet River National Wildlife Refuge |  |  |  |  |  | 1 |
| Great Meadows National Wildlife Refuge |  |  |  |  |  | 1 |
|  |  |  |  |  |  |  |
|  |  |  |  |  |  |  |
| **Region / State / NWRS Unit** | **Gas** | **Oil** | **Oil and Gas** | **Other** | **Total** | **Pipelines** |
| ***New Jersey*** |  |  |  |  |  | **6** |
| Cape May National Wildlife Refuge |  |  |  |  |  | 1 |
| Great Swamp National Wildlife Refuge |  |  |  |  |  | 4 |
| Wallkill River National Wildlife Refuge |  |  |  |  |  | 1 |
| ***New York*** |  |  |  | **1** | **1** | **5** |
| Montezuma National Wildlife Refuge |  |  |  | 1 | 1 | 5 |
| ***Pennsylvania*** | **2** |  |  |  | **2** | **8** |
| Erie National Wildlife Refuge | 2 |  |  |  | 2 | 1 |
| John Heinz National Wildlife Refuge At Tinicum |  |  |  |  |  | 7 |
| ***Virginia*** |  |  |  |  |  | **2** |
| Featherstone National Wildlife Refuge |  |  |  |  |  | 1 |
| Great Dismal Swamp National Wildlife Refuge |  |  |  |  |  | 1 |
| ***West Virginia*** | **5** | **4** | **11** | **27** | **47** | **2** |
| Canaan Valley National Wildlife Refuge | 4 |  |  | 3 | 7 | 1 |
| Ohio River Islands National Wildlife Refuge | 1 | 4 | 11 | 24 | 40 | 1 |
|  |  |  |  |  |  |  |
| **Mountain - Prairie Region (6)** | **42** | **37** | **2** | **41** | **122** | **60** |
| ***Colorado*** |  |  |  | **3** | **3** | **7** |
| Arapaho National Wildlife Refuge |  |  |  |  |  | 1 |
| Baca National Wildlife Refuge |  |  |  | 2 | 2 |  |
| Monte Vista National Wildlife Refuge |  |  |  |  |  | 1 |
| Rocky Flats National Wildlife Refuge |  |  |  |  |  | 2 |
| Rocky Mountain Arsenal National Wildlife Refuge |  |  |  | 1 | 1 | 3 |
| ***Kansas*** | **1** | **25** | **1** |  | **27** | **9** |
| Flint Hills National Wildlife Refuge |  |  |  |  |  | 7 |
| Quivira National Wildlife Refuge | 1 | 25 | 1 |  | 27 | 2 |
| ***Montana*** | **40** | **9** |  | **34** | **83** | **4** |
| Benton Lake National Wildlife Refuge |  |  |  | 2 | 2 |  |
| Benton Lake Wetland Management District | 4 |  |  | 7 | 11 | 1 |
| Bowdoin National Wildlife Refuge | 1 |  |  | 1 | 2 |  |
| Bowdoin Wetland Management District | 21 |  |  | 2 | 23 |  |
| Charles M. Russell National Wildlife Refuge |  |  |  |  |  | 2 |
| Hailstone National Wildlife Refuge |  |  |  | 1 | 1 |  |
| Halfbreed Lake National Wildlife Refuge | 1 |  |  | 4 | 5 |  |
| Hewitt Lake National Wildlife Refuge | 13 |  |  | 1 | 14 |  |
| Lake Mason National Wildlife Refuge |  |  |  | 2 | 2 |  |
| Medicine Lake National Wildlife Refuge |  | 2 |  | 3 | 5 |  |
| Northeast Montana Wetland Management District |  | 7 |  | 11 | 18 | 1 |
| ***Nebraska*** |  |  |  |  |  | **6** |
| Rainwater Basin Wetland Management District |  |  |  |  |  | 6 |
| **Region / State / NWRS Unit** | **Gas** | **Oil** | **Oil and Gas** | **Other** | **Total** | **Pipelines** |
| ***North Dakota*** |  |  | **1** |  | **1** | **16** |
| Audubon Wetland Management District |  |  |  |  |  | 2 |
| Chase Lake Wetland Management District |  |  |  |  |  | 2 |
| Devils Lake Wetland Management District |  |  |  |  |  | 5 |
| J. Clark Salyer Wetland Management District |  |  |  |  |  | 2 |
| Lake Ilo National Wildlife Refuge |  |  | 1 |  | 1 |  |
| Long Lake Wetland Management District |  |  |  |  |  | 1 |
| Sullys Hill National Game Preserve |  |  |  |  |  | 1 |
| Valley City Wetland Management District |  |  |  |  |  | 3 |
| ***South Dakota*** |  |  |  |  |  | **10** |
| Huron Wetland Management District |  |  |  |  |  | 2 |
| Lake Andes Wetland Management District |  |  |  |  |  | 1 |
| Madison Wetland Management District |  |  |  |  |  | 4 |
| Sand Lake Wetland Management District |  |  |  |  |  | 2 |
| Waubay Wetland Management District |  |  |  |  |  | 1 |
| ***Utah*** | **1** | **3** |  | **3** | **7** | **2** |
| Bear River Migratory Bird Refuge |  |  |  | 3 | 3 | 1 |
| Colorado River Wildlife Management Area | 1 | 2 |  |  | 3 |  |
| Ouray National Wildlife Refuge |  | 1 |  |  | 1 | 1 |
| ***Wyoming*** |  |  |  | **1** | **1** | **6** |
| Bamforth National Wildlife Refuge |  |  |  |  |  | 1 |
| Mortenson Lake National Wildlife Refuge |  |  |  | 1 | 1 | 1 |
| Pathfinder National Wildlife Refuge |  |  |  |  |  | 3 |
| Seedskadee National Wildlife Refuge |  |  |  |  |  | 1 |
|  |  |  |  |  |  |  |
| **Alaska Region (7)** | **75** | **66** | **4** | **54** | **199** | **7** |
| ***Alaska*** | **75** | **66** | **4** | **54** | **199** | **7** |
| Alaska Peninsula National Wildlife Refuge |  |  |  | 5 | 5 |  |
| Becharof National Wildlife Refuge |  |  |  | 2 | 2 |  |
| Kenai National Wildlife Refuge | 75 | 66 | 4 | 46 | 191 | 7 |
| Yukon Delta National Wildlife Refuge |  |  |  | 1 | 1 |  |
|  |  |  |  |  |  |  |
| **Pacific Southwest Region (8)** | **8** | **45** |  | **75** | **128** | **25** |
| ***California*** | **8** | **45** |  | **70** | **123** | **24** |
| Antioch Dunes National Wildlife Refuge |  |  |  |  |  | 1 |
| Bitter Creek National Wildlife Refuge |  |  |  | 12 | 12 | 1 |
| Butte Sink Wildlife Management Area |  |  |  | 1 | 1 |  |
| Cibola National Wildlife Refuge |  |  |  |  |  | 1 |
| Coachella Valley National Wildlife Refuge |  |  |  |  |  | 1 |
| Colusa National Wildlife Refuge | 1 |  |  | 3 | 4 |  |
| **Region / State / NWRS Unit** | **Gas** | **Oil** | **Oil and Gas** | **Other** | **Total** | **Pipelines** |
| Delevan National Wildlife Refuge | 1 |  |  | 7 | 8 |  |
| Don Edwards San Francisco Bay National Wildlife Refuge |  |  |  |  |  | 1 |
| Ellicott Slough National Wildlife Refuge |  |  |  |  |  | 1 |
| Grasslands Wildlife Management Area |  |  |  | 6 | 6 |  |
| Guadalupe-Nipomo Dunes National Wildlife Refuge |  | 2 |  | 1 | 3 |  |
| Havasu National Wildlife Refuge |  |  |  |  |  | 11 |
| Hopper Mountain National Wildlife Refuge |  | 17 |  | 2 | 19 |  |
| Humboldt Bay National Wildlife Refuge |  |  |  | 1 | 1 |  |
| Kern National Wildlife Refuge |  |  |  | 2 | 2 | 1 |
| Merced National Wildlife Refuge |  |  |  | 1 | 1 |  |
| North Central Valley Wildlife Management Area | 6 |  |  | 22 | 28 |  |
| Pixley National Wildlife Refuge |  |  |  | 1 | 1 | 1 |
| Sacramento River National Wildlife Refuge |  |  |  |  |  | 1 |
| San Joaquin River National Wildlife Refuge |  |  |  | 5 | 5 | 1 |
| San Pablo Bay National Wildlife Refuge |  |  |  | 1 | 1 |  |
| Seal Beach National Wildlife Refuge |  | 26 |  | 4 | 30 | 1 |
| Sutter National Wildlife Refuge |  |  |  |  |  | 2 |
| Tijuana Slough National Wildlife Refuge |  |  |  | 1 | 1 |  |
| ***Nevada*** |  |  |  | **5** | **5** | **1** |
| Fallon National Wildlife Refuge |  |  |  | 1 | 1 |  |
| Stillwater National Wildlife Refuge |  |  |  | 4 | 4 | 1 |
| **Grand Total** | **2201** | **971** | **59** | **1771** | **5002** | **595** |

Other - = includes wells other than oil and gas, such as injection, saltwater disposal, enhanced oil recovery, dry, observation, stratigraphic, other, and production type data not available (N/A)
