# Supplementary material for: Oil and Gas Wells and Pipelines on U.S. Wildlife Refuges: Challenges for Managers
Source: PLoS One. 2015 Apr 27;10(4):e0124085. doi: 10.1371/journal.pone.0124085 (PMC4410920; doi:10.1371/journal.pone.0124085)
Supplement: S2 Table — (DOCX) [file pone.0124085.s002.docx]

Table S2. Injection wells on NWRS units.

| **Region / NWRS Unit** | **Active** | **Inactive** | **P&A** | **Other Status** | **Total** |
| --- | --- | --- | --- | --- | --- |
| **Southwest Region (2)** | **112** | **21** | **15** | **13** | **161** |
| Anahuac National Wildlife Refuge | 2 |  | 2 |  | 4 |
| Aransas National Wildlife Refuge | 3 |  | 1 |  | 4 |
| Attwater Prairie Chicken National Wildlife Refuge |  | 1 |  |  | 1 |
| Brazoria National Wildlife Refuge |  | 1 |  |  | 1 |
| Caddo Lake National Wildlife Refuge | 1 |  |  | 1 | 2 |
| Deep Fork National Wildlife Refuge | 78 | 4 |  |  | 82 |
| Hagerman National Wildlife Refuge | 21 | 6 | 11 | 11 | 49 |
| Laguna Atascosa National Wildlife Refuge |  | 1 |  |  | 1 |
| Lower Rio Grande Valley National Wildlife Refuge | 5 | 4 | 1 |  | 10 |
| McFaddin National Wildlife Refuge | 2 | 2 |  |  | 4 |
| San Bernard National Wildlife Refuge |  | 2 |  | 1 | 3 |
|  |  |  |  |  |  |
| **Midwest Region (3)** | **1** | **4** |  |  | **5** |
| Patoka River National Wildlife Refuge | 1 | 4 |  |  | 5 |
|  |  |  |  |  |  |
| **Southeast Region (4)** | **5** | **1** | **20** | **7** | **33** |
| Felsenthal National Wildlife Refuge |  |  |  | 4 | 4 |
| St. Catherine Creek National Wildlife Refuge | 5 | 1 | 20 | 3 | 29 |
|  |  |  |  |  |  |
| **Mountain - Prairie Region (6)** |  | **2** |  |  | **2** |
| Northeast Montana Wetland Management District |  | 2 |  |  | 2 |
|  |  |  |  |  |  |
| **Alaska Region (7)** | **2** |  |  |  | **2** |
| Kenai National Wildlife Refuge | 2 |  |  |  | 2 |
|  |  |  |  |  |  |
| **Pacific Southwest Region (8)** | **1** | **1** |  | **1** | **3** |
| Seal Beach National Wildlife Refuge | 1 | 1 |  | 1 | 3 |
| **Grand Total** | **121** | **29** | **35** | **21** | **206** |

Active – includes injection wells that are injecting gas or fluids underground

Inactive – includes injection wells with a status ≠ active (e.g. inactive, plugged and abandoned, temporarily abandoned, shut-in, unknown)

P & A – Plugged and Abandoned

Other Status – temporarily abandoned, drilling, well status not available, or shut-in.
