# Supplementary material for: Oil and Gas Wells and Pipelines on U.S. Wildlife Refuges: Challenges for Managers
Source: PLoS One. 2015 Apr 27;10(4):e0124085. doi: 10.1371/journal.pone.0124085 (PMC4410920; doi:10.1371/journal.pone.0124085)
Supplement: S3 Table — [P&A = plugged and abandoned; N/A = status data not available; Other Status = includes other status codes not listed in table, such as dry hole, suspended, temporarily abandoned, unknown, or “blank” (i.e. not data)]. (DOCX) [file pone.0124085.s003.docx]

| **Region / NWRS Unit** | **Active** | **Inactive** | **P&A** | **Shut In** | **(N/A)** | **Other Status** | **Grand Total** |
| --- | --- | --- | --- | --- | --- | --- | --- |
| **Southwest Region (2)** | **338** | **328** | **48** |  | **203** | **57** | **974** |
| Anahuac National Wildlife Refuge | 4 | 7 | 4 |  |  |  | 15 |
| Aransas National Wildlife Refuge | 8 | 56 | 8 |  |  |  | 72 |
| Attwater Prairie Chicken National Wildlife Refuge | 10 | 10 |  |  |  |  | 20 |
| Big Boggy National Wildlife Refuge |  | 1 |  |  |  |  | 1 |
| Bitter Lake National Wildlife Refuge | 13 |  | 1 |  |  | 1 | 15 |
| Brazoria National Wildlife Refuge | 3 | 16 |  |  |  |  | 19 |
| Buenos Aires National Wildlife Refuge |  |  |  |  |  | 1 | 1 |
| Caddo Lake National Wildlife Refuge | 4 |  |  |  |  | 1 | 5 |
| Deep Fork National Wildlife Refuge | 149 | 60 |  |  | 164 | 27 | 400 |
| Hagerman National Wildlife Refuge | 50 | 43 | 12 |  |  | 11 | 116 |
| Laguna Atascosa National Wildlife Refuge | 1 | 7 | 3 |  |  | 0 | 11 |
| Little River National Wildlife Refuge | 1 |  |  |  | 1 | 6 | 8 |
| Lower Rio Grande Valley National Wildlife Refuge | 68 | 91 | 16 |  |  | 0 | 175 |
| McFaddin National Wildlife Refuge | 6 | 14 |  |  |  |  | 20 |
| Optima National Wildlife Refuge | 4 | 3 |  |  | 6 | 2 | 15 |
| Salt Plains National Wildlife Refuge | 3 | 1 |  |  | 8 | 1 | 13 |
| San Bernard National Wildlife Refuge | 13 | 14 | 2 |  |  | 1 | 30 |
| Sequoyah National Wildlife Refuge |  |  |  |  | 4 |  | 4 |
| Sevilleta National Wildlife Refuge |  |  |  |  | 2 |  | 2 |
| Texas Point National Wildlife Refuge |  | 2 |  |  |  |  | 2 |
| Tishomingo National Wildlife Refuge | 1 |  |  |  | 9 | 4 | 14 |
| Trinity River National Wildlife Refuge |  | 2 | 2 |  |  | 0 | 4 |
| Washita National Wildlife Refuge |  | 1 |  |  | 9 | 2 | 12 |

|  |  |  |  |  |  |  |  |
| --- | --- | --- | --- | --- | --- | --- | --- |
| **Region / NWRS Unit** | **Active** | **Inactive** | **P&A** | **Shut In** | **(N/A)** | **Other Status** | **Grand Total** |
| **Midwest Region (3)** | **16** | **83** |  |  |  | **3** | **102** |
| Big Muddy National Fish And Wildlife Refuge |  | 4 |  |  |  |  | 4 |
| Big Oaks National Wildlife Refuge |  | 5 |  |  |  |  | 5 |
| Kirtlands Warbler Wildlife Management Area | 1 | 1 |  |  |  |  | 2 |
| Muscatatuck National Wildlife Refuge |  | 1 |  |  |  |  | 1 |
| Patoka River National Wildlife Refuge | 15 | 72 |  |  |  | 3 | 90 |
|  |  |  |  |  |  |  |  |
| **Southeast Region (4)** | **1163** | **1697** | **469** | **17** | **75** | **6** | **3427** |
| Atchafalaya National Wildlife Refuge | 4 | 42 |  |  |  |  | 46 |
| Bald Knob National Wildlife Refuge |  |  |  |  | 3 |  | 3 |
| Bayou Cocodrie National Wildlife Refuge |  | 49 |  |  |  |  | 49 |
| Bayou Sauvage National Wildlife Refuge |  | 4 |  |  |  |  | 4 |
| Bayou Teche National Wildlife Refuge | 1 | 34 |  |  |  |  | 35 |
| Big Branch Marsh National Wildlife Refuge |  | 4 |  |  |  |  | 4 |
| Black Bayou Lake National Wildlife Refuge | 60 | 25 |  |  |  |  | 85 |
| Breton National Wildlife Refuge |  | 3 |  |  |  |  | 3 |
| Cache River National Wildlife Refuge |  |  |  |  | 2 |  | 2 |
| Cahaba River National Wildlife Refuge |  | 14 |  |  |  |  | 14 |
| Cameron Prairie National Wildlife Refuge |  | 14 |  |  |  |  | 14 |
| Cat Island National Wildlife Refuge |  | 5 |  |  |  |  | 5 |
| Catahoula National Wildlife Refuge | 6 | 69 |  |  |  |  | 75 |
| D'arbonne National Wildlife Refuge | 100 | 183 |  |  |  |  | 283 |
| Delta National Wildlife Refuge | 21 | 342 |  |  |  |  | 363 |
| Felsenthal National Wildlife Refuge |  |  |  |  | 57 |  | 57 |
| Florida Panther National Wildlife Refuge |  |  |  |  | 2 |  | 2 |
| Grand Bay National Wildlife Refuge |  |  | 1 |  |  |  | 1 |
| Grand Cote National Wildlife Refuge |  | 2 |  |  |  |  | 2 |
| **Region / NWRS Unit** | **Active** | **Inactive** | **P&A** | **Shut In** | **(N/A)** | **Other Status** | **Grand Total** |
| Lacassine National Wildlife Refuge | 5 | 75 |  |  |  |  | 80 |
| Lake Ophelia National Wildlife Refuge | 1 | 55 |  |  |  |  | 56 |
| Mandalay National Wildlife Refuge | 1 | 42 |  |  |  |  | 43 |
| Mississippi Sandhill Crane National Wildlife Refuge |  |  | 1 |  |  |  | 1 |
| National Key Deer Refuge |  |  |  |  | 1 |  | 1 |
| Overflow National Wildlife Refuge |  |  |  |  | 1 |  | 1 |
| Panther Swamp National Wildlife Refuge |  |  | 2 |  |  |  | 2 |
| Red River National Wildlife Refuge | 2 | 55 |  |  |  |  | 57 |
| Reelfoot National Wildlife Refuge |  |  |  |  | 1 |  | 1 |
| Sabine National Wildlife Refuge | 17 | 81 |  |  |  |  | 98 |
| St. Catherine Creek National Wildlife Refuge | 13 | 9 | 464 | 17 | 5 | 6 | 514 |
| Ten Thousand Islands National Wildlife Refuge |  |  |  |  | 1 |  | 1 |
| Tensas River National Wildlife Refuge | 4 | 108 |  |  |  |  | 112 |
| Upper Ouachita National Wildlife Refuge | 928 | 482 |  |  |  |  | 1410 |
| White River National Wildlife Refuge |  |  |  |  | 2 |  | 2 |
| Yazoo National Wildlife Refuge |  |  | 1 |  |  |  | 1 |
|  |  |  |  |  |  |  |  |
| **Northeast Region (5)** |  |  | **24** |  | **23** |  | **47** |
| Canaan Valley National Wildlife Refuge |  |  | 6 |  | 1 |  | 7 |
| Erie National Wildlife Refuge |  |  |  |  |  |  | 2 |
| Montezuma National Wildlife Refuge |  |  |  |  |  |  | 1 |
| Ohio River Islands National Wildlife Refuge |  |  | 18 |  | 22 |  | 40 |
|  |  |  |  |  |  |  |  |
| **Mountain - Prairie Region (6)** | **43** | **67** | **5** | **3** | **4** |  | **122** |
| Baca National Wildlife Refuge |  |  |  |  | 2 |  | 2 |
| Bear River Migratory Bird Refuge |  |  | 3 |  |  |  | 3 |
| Benton Lake National Wildlife Refuge |  | 2 |  |  |  |  | 2 |
| Benton Lake Wetland Management District | 3 | 8 |  |  |  |  | 11 |
| **Region / NWRS Unit** | **Active** | **Inactive** | **P&A** | **Shut In** | **(N/A)** | **Other Status** | **Grand Total** |
| Bowdoin National Wildlife Refuge | 1 | 1 |  |  |  |  | 2 |
| Bowdoin Wetland Management District | 18 | 5 |  |  |  |  | 23 |
| Colorado River Wildlife Management Area | 1 | 1 | 1 |  |  |  | 3 |
| Hailstone National Wildlife Refuge |  | 1 |  |  |  |  | 1 |
| Halfbreed Lake National Wildlife Refuge |  | 5 |  |  |  |  | 5 |
| Hewitt Lake National Wildlife Refuge | 9 | 5 |  |  |  |  | 14 |
| Lake Ilo National Wildlife Refuge | 1 |  |  |  |  |  | 1 |
| Lake Mason National Wildlife Refuge |  | 2 |  |  |  |  | 2 |
| Medicine Lake National Wildlife Refuge | 2 | 3 |  |  |  |  | 5 |
| Mortenson Lake National Wildlife Refuge |  |  | 1 |  |  |  | 1 |
| Northeast Montana Wetland Management District | 2 | 13 |  | 3 |  |  | 18 |
| Ouray National Wildlife Refuge |  |  |  |  | 1 |  | 1 |
| Quivira National Wildlife Refuge | 6 | 21 |  |  |  |  | 27 |
| Rocky Mountain Arsenal National Wildlife Refuge |  |  |  |  | 1 |  | 1 |
|  |  |  |  |  |  |  |  |
| **Alaska Region (7)** | **80** | **4** | **101** | **1** | **7** | **6** | **199** |
| Alaska Peninsula National Wildlife Refuge |  |  | 4 |  |  | 1 | 5 |
| Becharof National Wildlife Refuge |  |  | 2 |  |  |  | 2 |
| Kenai National Wildlife Refuge | 80 | 4 | 94 | 1 | 7 | 5 | 191 |
| Yukon Delta National Wildlife Refuge |  |  | 1 |  |  |  | 1 |
|  |  |  |  |  |  |  |  |
| **Pacific Southwest Region (8)** | **25** | **17** | **71** | **9** | **6** |  | **128** |
| Bitter Creek National Wildlife Refuge |  |  | 12 |  |  |  | 12 |
| Butte Sink Wildlife Management Area |  |  | 1 |  |  |  | 1 |
| Colusa National Wildlife Refuge |  |  | 4 |  |  |  | 4 |
| Delevan National Wildlife Refuge |  |  | 7 | 1 |  |  | 8 |
| Fallon National Wildlife Refuge |  |  |  |  | 1 |  | 1 |
| Grasslands Wildlife Management Area |  |  | 6 |  |  |  | 6 |
| **Region / NWRS Unit** | **Active** | **Inactive** | **P&A** | **Shut In** | **(N/A)** | **Other Status** | **Grand Total** |
| Guadalupe-Nipomo Dunes National Wildlife Refuge |  | 2 | 1 |  |  |  | 3 |
| Hopper Mountain National Wildlife Refuge | 13 |  | 2 | 3 | 1 |  | 19 |
| Humboldt Bay National Wildlife Refuge |  |  | 1 |  |  |  | 1 |
| Kern National Wildlife Refuge |  |  | 2 |  |  |  | 2 |
| Merced National Wildlife Refuge |  |  | 1 |  |  |  | 1 |
| North Central Valley Wildlife Management Area | 1 |  | 25 | 2 |  |  | 28 |
| Pixley National Wildlife Refuge |  |  | 1 |  |  |  | 1 |
| San Joaquin River National Wildlife Refuge |  |  | 5 |  |  |  | 5 |
| San Pablo Bay National Wildlife Refuge |  |  | 1 |  |  |  | 1 |
| Seal Beach National Wildlife Refuge | 11 | 15 | 1 | 3 |  |  | 30 |
| Stillwater National Wildlife Refuge |  |  |  |  | 4 |  | 4 |
| Tijuana Slough National Wildlife Refuge |  |  | 1 |  |  |  | 1 |
| **Grand Total** | **1665** | **2196** | **718** | **30** | **318** | **75** | **5002** |

Active – includes oil & gas wells that are producing oil and gas and other wells that are injecting gas or fluids underground

Inactive– includes all wells with a status ≠ active (e.g. inactive, plugged and abandoned, temporarily abandoned, shut-in, dry hole, unknown

Shut In - a well capable of production or injection by opening valves or powering equipment
