# Supplementary material for: Oil and Gas Wells and Pipelines on U.S. Wildlife Refuges: Challenges for Managers
Source: PLoS One. 2015 Apr 27;10(4):e0124085. doi: 10.1371/journal.pone.0124085 (PMC4410920; doi:10.1371/journal.pone.0124085)
Supplement: S4 Table — (DOCX) [file pone.0124085.s004.docx]

| **Region / NWRS Unit** | **Gas** | **Oil** | **Oil & Gas** | **Other** | **Grand Total** |
| --- | --- | --- | --- | --- | --- |
| **Southwest Region (2)** | **358** | **282** | **26** | **308** | **974** |
| **Anahuac National Wildlife Refuge** | **5** | **7** |  | **3** | **15** |
| Active |  | 3 |  | 1 | 4 |
| Inactive | 3 | 4 |  |  | 7 |
| P & A | 2 |  |  | 2 | 4 |
| **Aransas National Wildlife Refuge** | **58** | **8** | **4** | **2** | **72** |
| Active | 6 |  | 1 | 1 | 8 |
| Inactive | 45 | 8 | 3 |  | 56 |
| P & A | 7 |  |  | 1 | 8 |
| **Attwater Prairie Chicken National Wildlife Refuge** | **19** | **1** |  |  | **20** |
| Active | 10 |  |  |  | 10 |
| Inactive | 9 | 1 |  |  | 10 |
| **Big Boggy National Wildlife Refuge** | **1** |  |  |  | **1** |
| Inactive | 1 |  |  |  | 1 |
| **Bitter Lake National Wildlife Refuge** | **5** | **10** |  |  | **15** |
| Active | 4 | 9 |  |  | 13 |
| P & A | 1 |  |  |  | 1 |
| Temporarily Abandoned |  | 1 |  |  | 1 |
| **Brazoria National Wildlife Refuge** | **12** | **4** | **3** |  | **19** |
| Active | 3 |  |  |  | 3 |
| Inactive | 9 | 4 | 3 |  | 16 |
| **Buenos Aires National Wildlife Refuge** |  |  |  | **1** | **1** |
| Dry |  |  |  | 1 | 1 |
| **Caddo Lake National Wildlife Refuge** | **2** | **2** |  | **1** | **5** |
| Active | 2 | 2 |  |  | 4 |
| Temporarily Abandoned |  |  |  | 1 | 1 |
| **Deep Fork National Wildlife Refuge** | **26** | **147** |  | **227** | **400** |
| (N/A) | 16 | 48 |  | 100 | 164 |
| Active | 2 | 68 |  | 79 | 149 |
| Dry |  | 3 |  | 24 | 27 |
| Inactive | 8 | 28 |  | 24 | 60 |
| **Hagerman National Wildlife Refuge** | **3** | **71** | **4** | **38** | **116** |
| Active | 2 | 31 | 2 | 15 | 50 |
| Drilling |  |  |  | 1 | 1 |
| Inactive |  | 40 | 2 | 1 | 43 |
| P & A | 1 |  |  | 11 | 12 |
| Temporarily Abandoned |  |  |  | 10 | 10 |
| **Laguna Atascosa National Wildlife Refuge** | **11** |  |  |  | **11** |
| Active | 1 |  |  |  | 1 |
| Inactive | 7 |  |  |  | 7 |
| P & A | 3 |  |  |  | 3 |
| **Little River National Wildlife Refuge** |  | **1** |  | **7** | **8** |
| (N/A) |  |  |  | 1 | 1 |
| Active |  | 1 |  |  | 1 |
| Dry |  |  |  | 6 | 6 |

| **Region / NWRS Unit** | **Gas** | **Oil** | **Oil & Gas** | **Other** | **Grand Total** |
| --- | --- | --- | --- | --- | --- |
| **Lower Rio Grande Valley National Wildlife Refuge** | **139** | **23** | **10** | **3** | **175** |
| Active | 60 | 4 | 2 | 2 | 68 |
| Inactive | 64 | 19 | 8 |  | 91 |
| P & A | 15 |  |  | 1 | 16 |
| **McFaddin National Wildlife Refuge** | **14** | **4** |  | **2** | **20** |
| Active |  | 4 |  | 2 | 6 |
| Inactive | 14 |  |  |  | 14 |
| **Optima National Wildlife Refuge** | **13** |  |  | **2** | **15** |
| (N/A) | 6 |  |  |  | 6 |
| Active | 4 |  |  |  | 4 |
| Dry |  |  |  | 2 | 2 |
| Inactive | 3 |  |  |  | 3 |
| **Salt Plains National Wildlife Refuge** | **11** | **1** |  | **1** | **13** |
| (N/A) | 8 |  |  |  | 8 |
| Active | 3 |  |  |  | 3 |
| Dry |  |  |  | 1 | 1 |
| Inactive |  | 1 |  |  | 1 |
| **San Bernard National Wildlife Refuge** | **24** | **3** | **2** | **1** | **30** |
| Active | 9 | 3 | 1 |  | 13 |
| Inactive | 13 |  | 1 |  | 14 |
| P & A | 2 |  |  |  | 2 |
| Temporarily Abandoned |  |  |  | 1 | 1 |
| **Sequoyah National Wildlife Refuge** |  |  |  | **4** | **4** |
| (N/A) |  |  |  | 4 | 4 |
| **Sevilleta National Wildlife Refuge** |  |  |  | **2** | **2** |
| (N/A) |  |  |  | 2 | 2 |
| **Texas Point National Wildlife Refuge** |  |  | **2** |  | **2** |
| Inactive |  |  | 2 |  | 2 |
| **Tishomingo National Wildlife Refuge** | **2** |  |  | **12** | **14** |
| (N/A) | 1 |  |  | 8 | 9 |
| Active | 1 |  |  |  | 1 |
| Dry |  |  |  | 4 | 4 |
| **Trinity River National Wildlife Refuge** | **3** |  | **1** |  | **4** |
| Inactive | 1 |  | 1 |  | 2 |
| P & A | 2 |  |  |  | 2 |
| **Washita National Wildlife Refuge** | **10** |  |  | **2** | **12** |
| (N/A) | 9 |  |  |  | 9 |
| Dry |  |  |  | 2 | 2 |
| Inactive | 1 |  |  |  | 1 |
| **Region / NWRS Unit** | **Gas** | **Oil** | **Oil & Gas** | **Other** | **Grand Total** |
| **Midwest Region (3)** | **2** | **7** |  | **93** | **102** |
| **Big Muddy National Fish And Wildlife Refuge** |  |  |  | **4** | **4** |
| Inactive |  |  |  | 4 | 4 |
| **Big Oaks National Wildlife Refuge** |  |  |  | **5** | **5** |
| Inactive |  |  |  | 5 | 5 |

| **Region / NWRS Unit** | **Gas** | **Oil** | **Oil & Gas** | **Other** | **Grand Total** |
| --- | --- | --- | --- | --- | --- |
| **Kirtlands Warbler Wildlife Management Area** | **2** |  |  |  | **2** |
| Active | 1 |  |  |  | 1 |
| Inactive | 1 |  |  |  | 1 |
| **Muscatatuck National Wildlife Refuge** |  |  |  | **1** | **1** |
| Inactive |  |  |  | 1 | 1 |
| **Patoka River National Wildlife Refuge** |  | **7** |  | **83** | **90** |
| Blank (no data) |  | 1 |  | 2 | 3 |
| Active |  | 6 |  | 9 | 15 |
| Inactive |  |  |  | 72 | 72 |
| **Southeast Region (4)** | **1709** | **530** | **16** | **1172** | **3427** |
| **Atchafalaya National Wildlife Refuge** | **9** | **12** | **2** | **23** | **46** |
| Active | 1 | 3 |  |  | 4 |
| Inactive | 8 | 9 | 2 | 23 | 42 |
| **Bald Knob National Wildlife Refuge** | **3** |  |  |  | **3** |
| (N/A) | 3 |  |  |  | 3 |
| **Bayou Cocodrie National Wildlife Refuge** |  |  |  | **49** | **49** |
| Inactive |  |  |  | 49 | 49 |
| **Bayou Sauvage National Wildlife Refuge** |  |  |  | **4** | **4** |
| Inactive |  |  |  | 4 | 4 |
| **Bayou Teche National Wildlife Refuge** | **14** | **3** |  | **18** | **35** |
| Active | 1 |  |  |  | 1 |
| Inactive | 13 | 3 |  | 18 | 34 |
| **Big Branch Marsh National Wildlife Refuge** |  |  |  | **4** | **4** |
| Inactive |  |  |  | 4 | 4 |
| **Black Bayou Lake National Wildlife Refuge** | **78** |  |  | **7** | **85** |
| Active | 60 |  |  |  | 60 |
| Inactive | 18 |  |  | 7 | 25 |
| **Breton National Wildlife Refuge** |  |  |  | **3** | **3** |
| Inactive |  |  |  | 3 | 3 |
| **Cache River National Wildlife Refuge** | **2** |  |  |  | **2** |
| (N/A) | 2 |  |  |  | 2 |
| **Cahaba River National Wildlife Refuge** |  |  |  | **14** | **14** |
| Inactive |  |  |  | 14 | 14 |
| **Cameron Prairie National Wildlife Refuge** |  |  |  | **14** | **14** |
| Inactive |  |  |  | 14 | 14 |
| **Cat Island National Wildlife Refuge** | **2** |  |  | **3** | **5** |
| Inactive | 2 |  |  | 3 | 5 |
| **Catahoula National Wildlife Refuge** | **1** | **20** |  | **54** | **75** |
| Active |  | 6 |  |  | 6 |
| Inactive | 1 | 14 |  | 54 | 69 |
| **D'arbonne National Wildlife Refuge** | **183** |  |  | **100** | **283** |
| Active | 100 |  |  |  | 100 |
| Inactive | 83 |  |  | 100 | 183 |
| **Delta National Wildlife Refuge** | **52** | **214** | **11** | **86** | **363** |
| Active | 3 | 18 |  |  | 21 |
| Inactive | 49 | 196 | 11 | 86 | 342 |
| **Felsenthal National Wildlife Refuge** | **3** | **48** |  | **6** | **57** |
| (N/A) | 3 | 48 |  | 6 | 57 |

| **Region / NWRS Unit** | **Gas** | **Oil** | **Oil & Gas** | **Other** | **Grand Total** |
| --- | --- | --- | --- | --- | --- |
| **Florida Panther National Wildlife Refuge** |  |  |  | **2** | **2** |
| (N/A) |  |  |  | 2 | 2 |
| **Grand Bay National Wildlife Refuge** |  |  |  | **1** | **1** |
| P & A |  |  |  | 1 | 1 |
| **Grand Cote National Wildlife Refuge** |  |  |  | **2** | **2** |
| Inactive |  |  |  | 2 | 2 |
| **Lacassine National Wildlife Refuge** | **32** | **5** | **1** | **42** | **80** |
| Active | 3 | 2 |  |  | 5 |
| Inactive | 29 | 3 | 1 | 42 | 75 |
| **Lake Ophelia National Wildlife Refuge** |  | **5** |  | **51** | **56** |
| Active |  | 1 |  |  | 1 |
| Inactive |  | 4 |  | 51 | 55 |
| **Mandalay National Wildlife Refuge** | **13** | **5** |  | **25** | **43** |
| Active | 1 |  |  |  | 1 |
| Inactive | 12 | 5 |  | 25 | 42 |
| **Mississippi Sandhill Crane National Wildlife Refuge** |  |  |  | **1** | **1** |
| P & A |  |  |  | 1 | 1 |
| **National Key Deer Refuge** |  |  |  | **1** | **1** |
| (N/A) |  |  |  | 1 | 1 |
| **Overflow National Wildlife Refuge** |  | **1** |  |  | **1** |
| (N/A) |  | 1 |  |  | 1 |
| **Panther Swamp National Wildlife Refuge** |  |  |  | **2** | **2** |
| P & A |  |  |  | 2 | 2 |
| **Red River National Wildlife Refuge** | **6** | **24** |  | **27** | **57** |
| Active | 2 |  |  |  | 2 |
| Inactive | 4 | 24 |  | 27 | 55 |
| **Reelfoot National Wildlife Refuge** |  |  |  | **1** | **1** |
| (N/A) |  |  |  | 1 | 1 |
| **Sabine National Wildlife Refuge** | **22** | **23** | **2** | **51** | **98** |
| Active | 4 | 13 |  |  | 17 |
| Inactive | 18 | 10 | 2 | 51 | 81 |
| **St. Catherine Creek National Wildlife Refuge** | **8** | **130** |  | **376** | **514** |
| (N/A) |  | 3 |  | 2 | 5 |
| Active |  | 8 |  | 5 | 13 |
| Dry Hole |  |  |  | 2 | 2 |
| Inactive |  | 1 |  | 8 | 9 |
| P & A | 8 | 100 |  | 356 | 464 |
| Shut In |  | 17 |  |  | 17 |
| Suspended |  |  |  | 3 | 3 |
| Unknown |  | 1 |  |  | 1 |
| **Ten Thousand Islands National Wildlife Refuge** |  |  |  | **1** | **1** |
| (N/A) |  |  |  | 1 | 1 |
| **Tensas River National Wildlife Refuge** | **1** | **36** |  | **75** | **112** |
| Active |  | 4 |  |  | 4 |
| Inactive | 1 | 32 |  | 75 | 108 |

| **Region / NWRS Unit** | **Gas** | **Oil** | **Oil & Gas** | **Other** | **Grand Total** |
| --- | --- | --- | --- | --- | --- |
| **Upper Ouachita National Wildlife Refuge** | **1280** | **2** |  | **128** | **1410** |
| Active | 928 |  |  |  | 928 |
| Inactive | 352 | 2 |  | 128 | 482 |
| **White River National Wildlife Refuge** |  | **2** |  |  | **2** |
| (N/A) |  | 2 |  |  | 2 |
| **Yazoo National Wildlife Refuge** |  |  |  | **1** | **1** |
| P & A |  |  |  | 1 | 1 |
| **Northeast Region (5)** | **7** | **4** | **11** | **28** | **50** |
| **Canaan Valley National Wildlife Refuge** | **4** |  |  | **3** | **7** |
| (N/A) | 1 |  |  |  | 1 |
| P & A | 3 |  |  | 3 | 6 |
| **Erie National Wildlife Refuge** | **2** |  |  |  | **2** |
| Va (not defined) | 2 |  |  |  | 2 |
| **Montezuma National Wildlife Refuge** |  |  |  | **1** | **1** |
| Unknown |  |  |  | 1 | 1 |
| **Ohio River Islands National Wildlife Refuge** | **1** | **4** | **11** | **24** | **40** |
| (N/A) |  | 2 | 3 | 17 | 22 |
| P & A | 1 | 2 | 8 | 7 | 18 |
| **Mountain - Prairie Region (6)** | **42** | **37** | **2** | **41** | **122** |
| **Baca National Wildlife Refuge** |  |  |  | **2** | **2** |
| (N/A) |  |  |  | 2 | 2 |
| **Bear River Migratory Bird Refuge** |  |  |  | **3** | **3** |
| P & A |  |  |  | 3 | 3 |
| **Benton Lake National Wildlife Refuge** |  |  |  | **2** | **2** |
| Inactive |  |  |  | 2 | 2 |
| **Benton Lake Wetland Management District** | **4** |  |  | **7** | **11** |
| Active | 3 |  |  |  | 3 |
| Inactive | 1 |  |  | 7 | 8 |
| **Bowdoin National Wildlife Refuge** | **1** |  |  | **1** | **2** |
| Active | 1 |  |  |  | 1 |
| Inactive |  |  |  | 1 | 1 |
| **Bowdoin Wetland Management District** | **21** |  |  | **2** | **23** |
| Active | 18 |  |  |  | 18 |
| Inactive | 3 |  |  | 2 | 5 |
| **Colorado River Wildlife Management Area** | **1** | **2** |  |  | **3** |
| Active |  | 1 |  |  | 1 |
| Inactive |  | 1 |  |  | 1 |
| P & A | 1 |  |  |  | 1 |
| **Hailstone National Wildlife Refuge** |  |  |  | **1** | **1** |
| Inactive |  |  |  | 1 | 1 |
| **Halfbreed Lake National Wildlife Refuge** | **1** |  |  | **4** | **5** |
| Inactive | 1 |  |  | 4 | 5 |
| **Hewitt Lake National Wildlife Refuge** | **13** |  |  | **1** | **14** |
| Active | 9 |  |  |  | 9 |
| Inactive | 4 |  |  | 1 | 5 |
| **Lake Ilo National Wildlife Refuge** |  |  | **1** |  | **1** |
| Active |  |  | 1 |  | 1 |
| **Lake Mason National Wildlife Refuge** |  |  |  | **2** | **2** |
| Inactive |  |  |  | 2 | 2 |
| **Region / NWRS Unit** | **Gas** | **Oil** | **Oil & Gas** | **Other** | **Grand Total** |
| **Medicine Lake National Wildlife Refuge** |  | **2** |  | **3** | **5** |
| Active |  | 2 |  |  | 2 |
| Inactive |  |  |  | 3 | 3 |
| **Mortenson Lake National Wildlife Refuge** |  |  |  | **1** | **1** |
| P & A |  |  |  | 1 | 1 |
| **Northeast Montana Wetland Management District** |  | **7** |  | **11** | **18** |
| Active |  | 2 |  |  | 2 |
| Inactive |  | 2 |  | 11 | 13 |
| Shut In |  | 3 |  |  | 3 |
| **Ouray National Wildlife Refuge** |  | **1** |  |  | **1** |
| (N/A) |  | 1 |  |  | 1 |
| **Quivira National Wildlife Refuge** | **1** | **25** | **1** |  | **27** |
| Active |  | 6 |  |  | 6 |
| Inactive | 1 | 19 | 1 |  | 21 |
| **Rocky Mountain Arsenal National Wildlife Refuge** |  |  |  | **1** | **1** |
| (N/A) |  |  |  | 1 | 1 |
| **Alaska Region (7)** | **75** | **66** | **4** | **54** | **199** |
| **Alaska Peninsula National Wildlife Refuge** |  |  |  | **5** | **5** |
| P & A |  |  |  | 4 | 4 |
| Suspended |  |  |  | 1 | 1 |
| **Becharof National Wildlife Refuge** |  |  |  | **2** | **2** |
| P & A |  |  |  | 2 | 2 |
| **Kenai National Wildlife Refuge** | **75** | **66** | **4** | **46** | **191** |
| (N/A) | 4 |  |  | 3 | 7 |
| Active | 39 | 37 | 1 | 3 | 80 |
| Inactive |  |  |  | 4 | 4 |
| P & A | 31 | 27 | 1 | 35 | 94 |
| Shut In |  | 1 |  |  | 1 |
| Suspended | 1 | 1 | 2 | 1 | 5 |
| **Yukon Delta National Wildlife Refuge** |  |  |  | **1** | **1** |
| P & A |  |  |  | 1 | 1 |
| **Pacific Southwest Region (8)** | **8** | **45** |  | **75** | **128** |
| **Bitter Creek National Wildlife Refuge** |  |  |  | **12** | **12** |
| P & A |  |  |  | 12 | 12 |
| **Butte Sink Wildlife Management Area** |  |  |  | **1** | **1** |
| P & A |  |  |  | 1 | 1 |
| **Colusa National Wildlife Refuge** | **1** |  |  | **3** | **4** |
| P & A | 1 |  |  | 3 | 4 |
| **Delevan National Wildlife Refuge** | **1** |  |  | **7** | **8** |
| P & A |  |  |  | 7 | 7 |
| Shut In | 1 |  |  |  | 1 |
| **Fallon National Wildlife Refuge** |  |  |  | **1** | **1** |
| (N/A) |  |  |  | 1 | 1 |
| **Grasslands Wildlife Management Area** |  |  |  | **6** | **6** |
| P & A |  |  |  | 6 | 6 |

| **Region / NWRS Unit** | **Gas** | **Oil** | **Oil & Gas** | **Other** | **Grand Total** |
| --- | --- | --- | --- | --- | --- |
| **Guadalupe-Nipomo Dunes National Wildlife Refuge** |  | **2** |  | **1** | **3** |
| Inactive |  | 2 |  |  | 2 |
| P & A |  |  |  | 1 | 1 |
| **Hopper Mountain National Wildlife Refuge** |  | **17** |  | **2** | **19** |
| (N/A) |  | 1 |  |  | 1 |
| Active |  | 13 |  |  | 13 |
| P & A |  |  |  | 2 | 2 |
| Shut In |  | 3 |  |  | 3 |
| **Humboldt Bay National Wildlife Refuge** |  |  |  | **1** | **1** |
| P & A |  |  |  | 1 | 1 |
| **Kern National Wildlife Refuge** |  |  |  | **2** | **2** |
| P & A |  |  |  | 2 | 2 |
| **Merced National Wildlife Refuge** |  |  |  | **1** | **1** |
| P & A |  |  |  | 1 | 1 |
| **North Central Valley Wildlife Management Area** | **6** |  |  | **22** | **28** |
| Active | 1 |  |  |  | 1 |
| P & A | 3 |  |  | 22 | 25 |
| Shut In | 2 |  |  |  | 2 |
| **Pixley National Wildlife Refuge** |  |  |  | **1** | **1** |
| P & A |  |  |  | 1 | 1 |
| **San Joaquin River National Wildlife Refuge** |  |  |  | **5** | **5** |
| P & A |  |  |  | 5 | 5 |
| **San Pablo Bay National Wildlife Refuge** |  |  |  | **1** | **1** |
| P & A |  |  |  | 1 | 1 |
| **Seal Beach National Wildlife Refuge** |  | **26** |  | **4** | **30** |
| Active |  | 10 |  | 1 | 11 |
| Inactive |  | 14 |  | 1 | 15 |
| P & A |  |  |  | 1 | 1 |
| Shut In |  | 2 |  | 1 | 3 |
| **Stillwater National Wildlife Refuge** |  |  |  | **4** | **4** |
| (N/A) |  |  |  | 4 | 4 |
| **Tijuana Slough National Wildlife Refuge** |  |  |  | **1** | **1** |
| P & A |  |  |  | 1 | 1 |
| **Grand Total** | **2201** | **971** | **58** | **1771** | **5002** |

Active – includes oil & gas wells that are producing oil and gas and other wells that are injecting gas or fluids underground

Inactive– includes all wells with a status ≠ active (e.g. inactive, plugged and abandoned, temporarily abandoned, shut-in, dry hole, unknown

Shut In - a well capable of production or injection by opening valves or powering equipment

P & A – Plugged and Abandoned

N/A – well status not available

Dry or Dry Hole – well not producing oil or gas following well completion

Suspended – well capped off temporarily

Unknown – status unknown
